# Modeling the Dynamics of Let-7-Coupled Gene Regulatory Networks Linking Cell Proliferation to Malignant Transformation

**DOI:** 10.3389/fphys.2019.00848

**Published:** 2019-07-11

**Authors:** Claude Gérard, Frédéric Lemaigre, Didier Gonze

**Affiliations:** ^1^de Duve Institute, Université catholique de Louvain, Brussels, Belgium; ^2^Unité de Chronobiologie Théorique, Faculté des Sciences, Université Libre de Bruxelles, Brussels, Belgium

**Keywords:** Cdk network, cell cycle, inflammatory circuit, deterministic model, cell population, random switch

## Abstract

Let-7 microRNA controls the expression of proteins that belong to two distinct gene regulatory networks, namely, a cyclin-dependent kinase (Cdk) network driving the cell cycle and a cell transformation network that can undergo an epigenetic switch between a non-transformed and a malignant transformed cell state. Using mathematical modeling and transcriptomic data analysis, we here investigate how Let-7 controls the Cdk-dependent cell cycle network, and how it couples the latter with the transformation network. We also assess the consequence of this coupling on cancer progression. Our analysis shows that the switch from a quiescent to a proliferative state depends on the relative levels of Let-7 and several cell cycle activators. Numerical simulations further indicate that the Let-7-coupled cell cycle and transformation networks mutually control each other, and our model identifies key players for this mutual control. Transcriptomic data analysis from The Cancer Genome Atlas (TCGA) suggests that the two networks are activated in cancer, in particular in gastrointestinal cancers, and that the activation levels vary significantly among patients affected by a same cancer type. Our mathematical model, when applied to a heterogeneous cell population, suggests that heterogeneity among tumors may in part result from stochastic switches between a non-transformed cell state with low proliferative capability and a transformed cell state with high proliferative property. The model further predicts that Let-7 may reduce tumor heterogeneity by decreasing the occurrence of stochastic switches toward a transformed, proliferative cell state. In conclusion, we identified the key components responsible for the qualitative dynamics of two networks interconnected by Let-7. The two networks are heterogeneously activated in several cancers, thereby stressing the need to consider patient’s specific characteristics to optimize therapeutic strategies.

## Introduction

Let-7 microRNAs control two distinct gene regulatory networks (GRNs) that regulate cell cycling and malignant transformation of breast cancer cells (Johnson et al., 2007; Iliopoulos et al., 2009). A cyclin-dependent kinase (Cdk) network controls the correct progression of the cell cycle along the G1, S, G2, and M phases (Morgan, 2007). Growth factors (GFs) and E2F stimulate, while Let-7 down-regulates the expression of several components of this Cdk-dependent cell cycle network (Bueno and Malumbres, 2011). Mathematical models focusing on post-translational regulations of cyclin/Cdk complexes were proposed to account for the dynamics of the Cdk network in mammals (Novak and Tyson, 2004; Gérard and Goldbeter, 2009). However, to our knowledge, no model has been proposed to study the impact of miRNAs on this network.

Let-7 is also a key component of a GRN that promotes cell transformation in response to an inflammatory stimulus (Iliopoulos et al., 2009). This GRN is characterized by a positive feedback loop (PFL), where a transient inflammatory stimulus is sufficient to induce the cells to undergo a PFL-dependent epigenetic switch from a non-transformed state toward a permanently malignant transformed state. We previously proposed a model describing the dynamics of this transformation GRN (Gérard et al., 2014). Our model suggested that a transient inflammatory signal induces an irreversible bistable switch in the expression of the GRN components, eventually leading to a stable epigenetic state, allowing cells to display increased motility and invasiveness. In this GRN, Let-7 prevents cell transformation by inhibiting the translation of interleukin-6 (IL6) and Ras, two oncogenic drivers.

Let-7 being a component common to the cell cycle and transformation networks, we now raise the following questions: how does Let-7 control the Cdk-dependent cell cycle network? Does Let-7 play a coupling role between the cell cycle GRN and the transformation GRN, and can the two GRNs be combined into a larger network that impacts on cancer progression? We address these issues using experiment-based mathematical modeling of the GRNs and by analyzing transcriptomic data from The Cancer Genome Atlas (TCGA). We characterize the qualitative dynamics resulting from the coupling between the two GRNs and show that their activation is cancer-specific and heterogeneous from patient to patient, stressing the need to consider patient’s specific characteristics.

## Results

### Structure of the Cell Cycle and Transformation Networks and Model Description

**FIGURE 1:**
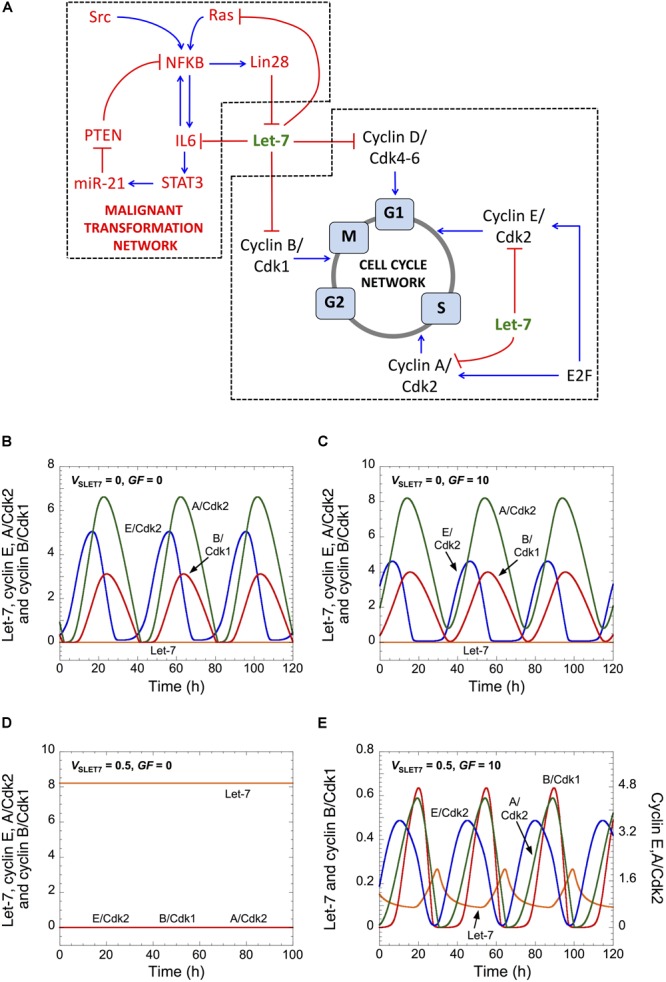
Let-7 and GF control cell cycle progression. **(A)** Scheme of both GRNs coupled by Let-7. **(B–E)** Temporal evolution of Let-7, cyclin E/Cdk2, cyclin A/Cdk2, and cyclin B/Cdk1 is shown in the absence (*V*_SLET7_ = 0 in panels **B,C**) or in the presence of Let-7 (*V*_SLET7_ = 0.5 in panels **D,E**), and in the absence (*GF* = 0 in panels **B,D**) or in the presence of GF (*GF* = 10 in panels **C,E**). Parameter values are as in Supplementary Table S5. A detailed scheme of both GRNs that includes all regulations considered in the model is in Supplementary Figure S1.

The structure of the Cdk network gives rise to a transient and sequential activation of the various cyclin/Cdk complexes, allowing for a correct progression through the different cell cycle phases (Figure 1A; Morgan, 2007; Gérard and Goldbeter, 2009). The activity of cyclin D/Cdk4-6 ensures the transition G0/G1 and the progression in G1. Cyclin E/Cdk2 promotes the G1/S transition, while cyclin A/Cdk2 elicits progression in S and G2. Finally, cyclin B/Cdk1 brings about G2/M transition and the entry of cell into mitosis (Figure 1A). In the model, Let-7 represses each cyclin/Cdk complex (see next section for details).

Let-7 is also at the core of a PFL in a malignant transformation network (Iliopoulos et al., 2009). Indeed, a transient inflammatory signal mediated by the oncoprotein Src activates NF-κB, which promotes Lin28, IL6, and STAT3 activation (Iliopoulos et al., 2010; Fofaria and Srivastava, 2015). Multiple links exist between cell proliferation and the processes of cell transformation and cell migration (Zhang and Liu, 2002). Indeed, NF-κB, Lin28, Ras, and STAT3 may all regulate cell proliferation, which strengthens the coupling between cell proliferation and cell transformation (Coqueret and Gascan, 2000; Bienvenu et al., 2001; Brantley et al., 2001; Ding et al., 2008; Xiong et al., 2008; Xu et al., 2009). Here, we do not aim to propose a comprehensive mathematical model to analyze the multiple, redundant, intertwined links between both GRNs. We focus on Let-7 and explore its potential role to couple both GRNs driving proliferation and malignant transformation.

The model is described by a set of 15 kinetic equations for the Cdk network driving the mammalian cell cycle and 14 kinetic equations for the inflammatory circuit that controls the dynamics of malignant transformation (Gérard et al., 2014). Each equation represents the temporal evolution of the expression level of one component of the network. For the cell cycle network, it includes the mRNAs of cyclin D, E, A, and B; the active form of E2F; the various cyclin/Cdk complexes (cyclin D/Cdk4-6, cyclin E/Cdk2, cyclin A/Cdk2 and cyclin B/Cdk1); and the active form of the anaphase-promoting complex (APC), which triggers degradation of cyclins A and B at the end of mitosis (Supplementary Figure S1). We assume that the different complexes cyclin/Cdk are formed immediately after the synthesis of cyclin, Cdk being present in excess, and consequently, we only consider the synthesis and degradation of these complexes. Variables are defined in Supplementary Table S1, the kinetic equations are given in Supplementary Table S3, detailed kinetic reactions of the mathematical model is given in Supplementary Table S4, and the parameters are defined in Supplementary Table S5.

### Relative Levels of Let-7 and Growth Factors Control Cell Cycling

To analyze the impact of Let-7 on the Cdk network dynamics, we built a qualitative skeleton mathematical model of cell cycle regulation by Let-7 (see Figure 1A for an overview of the model’s structure and Supplementary Figure S1 for a detailed description highlighting all regulatory interactions included in the model). The model is an extension of an earlier model of the Cdk network that accounted for the dynamics of the mammalian cell cycle (Gérard and Goldbeter, 2011). It now explicitly incorporates the mRNA form of each cyclin, enabling us to describe Let-7-mediated post-transcriptional regulation of cyclin synthesis. Let-7 represses the synthesis of multiple activators of the cell cycle, such as cyclins D, E, A, B, Cdk6, and E2F (see Bueno and Malumbres, 2011; Supplementary Table S2). For the sake of simplicity, we consider that Let-7 directly represses the translation of cyclins D, E, A, and B, by forming an “inactive” complex with their respective mRNA (once bound to Let-7, cyclin mRNA cannot be translated). In addition, GFs promote the synthesis of cyclin D, eliciting the G0/G1 transition and the entry of the cell into the cell cycle, while E2F activates synthesis of cyclins E and A. In this version of the model for the cell cycle, we do not consider the inhibitory impact of RB on cell cycle progression (Giacinti and Giordano, 2006; Gérard and Goldbeter, 2009).

As a consequence of its regulatory structure, the network self-organizes with sustained oscillations in the activity of the various cyclin/Cdk complexes, which correspond to successive rounds of cell cycling. The occurrence of the oscillations, however, depends on the levels of Let-7 and GF. In the absence of both Let-7 and GF, cells proliferate and sustained oscillations of the various cyclin/Cdk complexes develop (Figure 1B). This situation bears similarity with transformed or cancer cells, which are often characterized by down-regulation of Let-7 and signal-independent growth (Sotiropoulou et al., 2009; Hanahan and Weinberg, 2011). Starting from that condition, an increase in GF maintains cell proliferation (Figure 1C), while an increase in Let-7 suppresses cell proliferation (Johnson et al., 2007). The latter case is characterized by a stable steady state, with low levels of each cyclin/Cdk (Figure 1D). Finally, starting from that steady state, an increase in GF permits the recovery of cell proliferation (Figure 1E).

In this version of the model for the cell cycle, Let-7 is the only break for cell cycle progression. Thus, in the absence of Let-7, the Cdk network is able to enter into a sustained oscillatory regime in a GF-independent manner. Because there is a balance between inhibitors and activators of the cell cycle that controls the decision between quiescence and proliferation (Gérard and Goldbeter, 2014), an increase in the level of Let-7 renders the Cdk network dependent on an activator of the cell cycle, here GF, to enter into a sustained oscillatory regime. A previous theoretical study already predicted that a balance between E2F (activator of the cell cycle) and RB (inhibitor of the cell cycle) controls the existence of sustained oscillations of the Cdk network that are either dependent or independent on GF (Gérard and Goldbeter, 2012).

**FIGURE 2:**
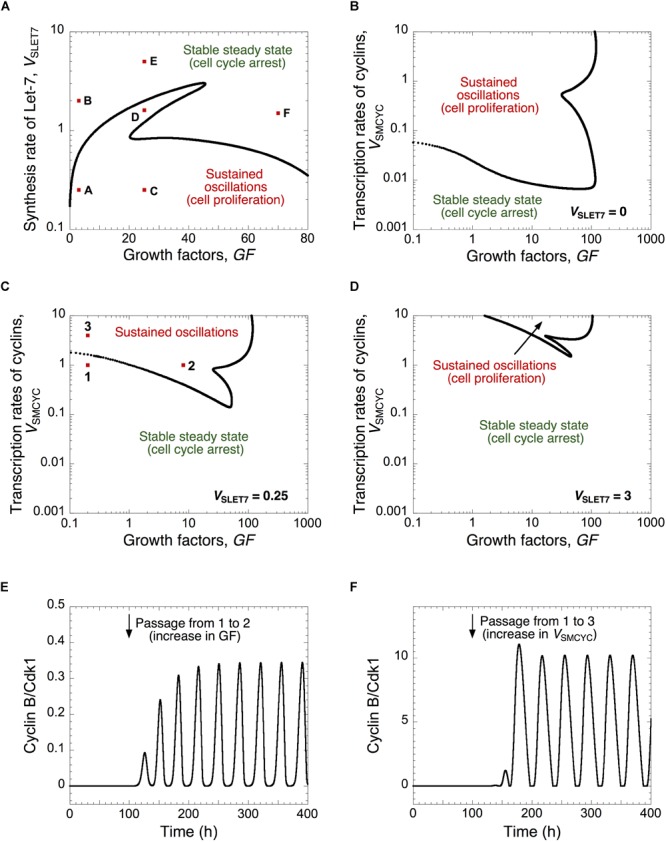
A balance between Let-7 and cell cycle activators determines the quiescence and proliferative state. **(A)** The Cdk network dynamics, i.e., sustained oscillations *versus* stable steady states, is represented in a two-parameter plane defined by the synthesis rate of Let-7, *V*_SLET7_, and the levels of GF. The Cdk network dynamics are indicated in a two-parameter plot as a function of the synthesis rate of the different cyclins, *V*_SMCYC_, and the levels of *GF* **(B)** in the absence of Let-7, *V*_SLET7_ = 0, **(C)** in the presence of intermediate, *V*_SLET7_ = 0.25, or **(D)** high Let-7 levels, *V*_SLET7_ = 3. **(E,F)** Temporal evolution of cyclin B/Cdk1 in the presence of GF overexpression (panel E where *GF* changes from 0.2 to 8, at *t* = 100 h, which corresponds to the switch from conditions 1 to 2 in panel **(C)**, or in the presence of cyclin overexpression (panel **F** where *V*_SMCYC_ changes from 1 to 4, at *t* = 100 h, which corresponds to the switch from 1 to 3 in panel **(C)**. Temporal evolution of cyclin E/Cdk2 and cyclin B/Cdk1 corresponding to conditions A to F in panel **(A)** are shown in Supplementary Figure S2. Other parameter values are as in Supplementary Table S5.

The respective impact of Let-7 and GFs can be visualized in a 2-parameter plane where the dynamical behavior of the cell cycle network is represented as a function of the synthesis rate of Let-7, *V*_SLET7_, and the level of growth factors, *GF* (Figure 2A). For large values of *V*_SLET7_ (high levels of Let-7), the Cdk network tends to a stable steady state corresponding to cell cycle arrest regardless of GF levels. This corroborates experimental results showing that Let-7 represses cell proliferation (Johnson et al., 2007; Zhu et al., 2015). In contrast, for small values of *V*_SLET7_ (low levels of Let-7), the cell cycle network is characterized by sustained oscillations. The temporal evolution of cyclin E/Cdk2 and cyclin B/Cdk1 corresponding to conditions A to F in Figure 2A are represented in Supplementary Figures S2A–F, respectively.

Note that we interpret the amplitude of the cyclin/Cdk oscillations in a semi-quantitative manner. We consider that the high amplitude of cyclin E/Cdk2 and cyclin B/Cdk1 oscillations enables cell proliferation by triggering G1/S and G2/M transitions, respectively (Figure 1, 2 and Supplementary Figures S2A,C). The sustained oscillations in cyclin E/Cdk2 with oscillations in cyclin B/Cdk1 of very small amplitude might correspond to endoreplication (Edgar et al., 2014) where multiple rounds of DNA replication occur without entry into mitosis (see temporal evolution in Supplementary Figure S2D, which corresponds to condition D in Figure 2A). Previous theoretical studies already showed that the regulatory structure of the cell cycle network in mammals is capable of generating endocycles (Gérard and Goldbeter, 2009). Near the Hopf bifurcation, the oscillations of the various cyclin/Cdk may not have sufficient amplitude to trigger the transitions into the successive cell cycle phases. However, the domain of limit-cycle oscillations nevertheless provides an estimate of the extent of the proliferation domain and how it is affected upon changes in parameter values; it also indicates what would be the impact of changes in control parameters.

The dynamics of the cell cycle network is further illustrated for different levels of Let-7 in a two-parameter plane defined by the synthesis rate of the cyclin/Cdk complexes, *V*_SMCYC_, and the level of GF (Figure 2B–D). By increasing Let-7, the domain of sustained oscillations, corresponding to cell proliferation, is reduced and limited to higher levels of cyclin/Cdk complexes (compare Figure 2B–D where *V*_SLET7_ is equal to 0, 0.25, and 3, respectively). From a stable steady state, corresponding to quiescence (condition 1 in Figure 2C), an increase in GF or an increase in the cyclin/Cdk levels may trigger the switch to sustained oscillations (see temporal evolution of cyclin B/Cdk1 in Figure 2E,F).

We concluded that progression or arrest of the cell cycle is controlled by the relative levels of Let-7 and GF, or of Let-7 and the cyclin/Cdk complexes. Thus, when designing efficient anti-cancer strategies, the model stresses the importance to consider the relative, rather than the absolute, expression levels of network components displaying opposing effects on cell cycling.

### Let-7 Couples the Cell Cycle and Transformation Networks

Let-7 belongs to both the Cdk-dependent cell cycle GRN and the malignant transformation network (Figure 1A). Therefore, we here determine the qualitative role of Let-7 as a coupling factor between the two GRNs, by analyzing the mutual impact of the GRNs on their respective dynamics.

**FIGURE 3:**
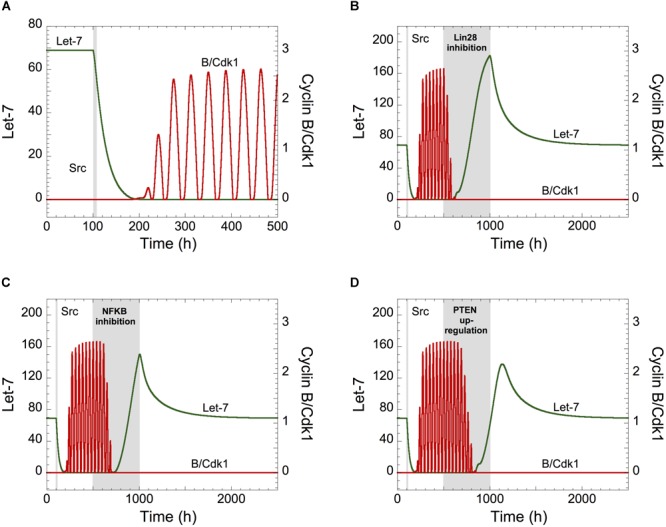
Let-7 is a central component between the proliferation and a malignant transformation network. **(A)** Effect of the inflammatory circuit on the Cdk network dynamics. **(A–D)** Starting from a non-transformed, quiescent, cell state, defined by high Let-7 and low cyclin B/Cdk1 levels, a transient increase in Src from *t* = 100 h to *t* = 105 h (gray area) triggers an epigenetic switch and cell proliferation characterized by Let-7 down-regulation and sustained oscillations of cyclin B/Cdk1. From that transformed, cell proliferative state, the temporal evolution of Let-7 and cyclin B/Cdk1 is illustrated in the presence of transient **(B)** Lin28 inhibition (*V*_SLIN28_ passes from 0.1 to 0 for 500 h < *t* < 1000 h), **(C)** NF-κB inhibition (*k*_AA1NFKB_ = *k*_AA2NFKB_ = *k*_AA3NFKB_ = 0 for 500 h < *t* < 1000 h), or **(D)** PTEN over-expression (*V*_SMPTEN_ passes from 0.001 to 10 for 500 h < *t* < 1000 h). Other parameter values are as in Supplementary Table S5.

Starting from a non-transformed, quiescent, cell state, defined by high Let-7 and low cyclin B/Cdk1 levels, a transient Src signal induced by inflammation triggers a down-regulation of Let-7, eliciting the switch of the cell cycle network from a stable steady state to sustained oscillations (Figure 3A). Thus, transient inflammatory signals can promote persistent cell proliferation. On the opposite, as observed in the experiments (Iliopoulos et al., 2009), starting from a transformed and proliferative cell state, transient inhibition of Lin28 or NF-κB or transient overexpression of PTEN durably impedes cell cycle progression (Figure 3B–D).

We concluded that transient modifications in the expression of the components of the inflammation-dependent bistable transformation network can impact the long-term behavior of the cell cycling network when Let-7 couples the two GRNs.

**FIGURE 4:**
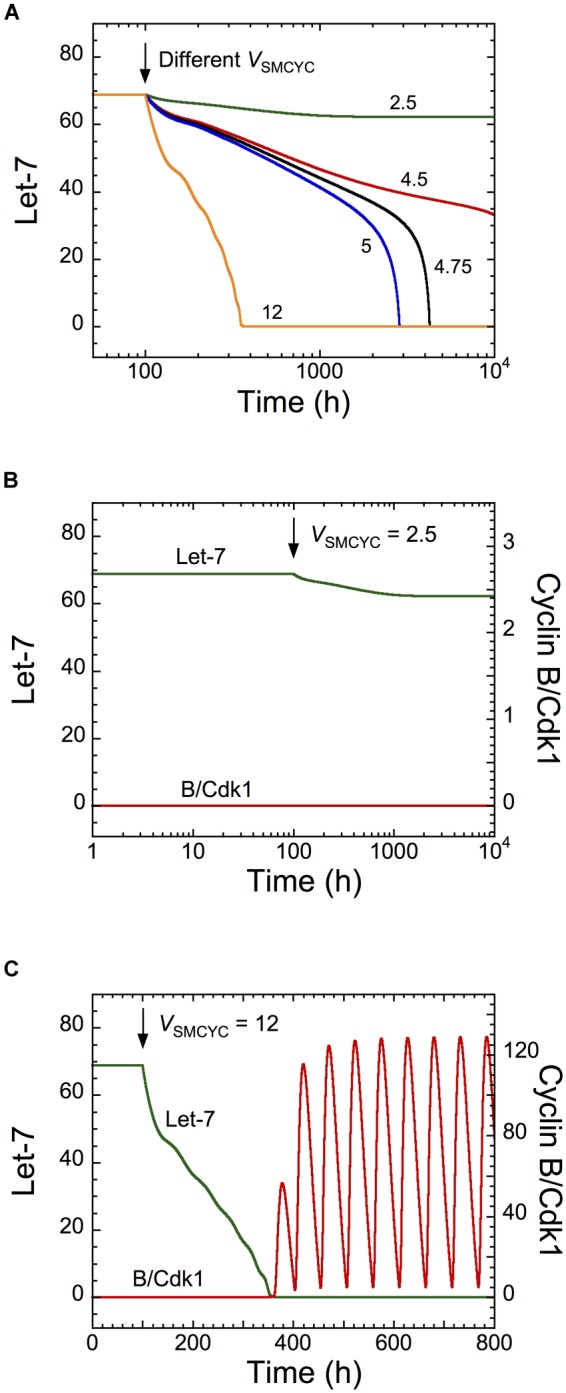
Overexpression of cyclins is predicted to impact on the dynamics of the transformation network. Temporal evolution of **(A)** Let-7, or **(B,C)** Let-7 and cyclin B/Cdk1 illustrated for different synthesis rates of cyclins, *V*_SMCYC_. From a non-transformed and quiescent cell state defined by high Let-7 levels and low levels of cyclin B/Cdk1, a sufficient increase in *V*_SMCYC_ (for *t* > 100 h) down-regulates Let-7, inducing a switch to a transformed and cell proliferative state. Parameter values are as in Supplementary Table S5.

To determine if the cell cycle network can modulate the dynamics of the transformation network, we simulated overexpression of all cyclins by increasing their synthesis rates, *via* the parameter *V*_SMCYC_. Cyclin overexpression promotes uncontrolled cell proliferation of cancer cells (Gillett et al., 1994; Pok et al., 2013). Starting from a non-transformed cell state (high Let-7 levels), the model shows a down-regulation of Let-7 when *V*_SMCYC_ increases (Figure 4A, where *V*_SMCYC_ increases at *t* = 100 h). The corresponding temporal evolution of Let-7 and cyclin B/Cdk1 is represented for different synthesis rates of cyclins (at *t* = 100 h, *V*_SMCYC_ changes from 1.5 to 2.5 in Figure 4B and from 1.5 to 12 in Figure 4C). For weak overexpression of cyclins, the non-transformed quiescent cell state is maintained, while for stronger cyclin overexpression, both GRNs are activated, leading to down-regulation of Let-7 and sustained oscillations of the cell cycle network (Figure 4B,C). Note that for mild overexpression of cyclin, the time needed for Let-7 down-regulation is rather long, possibly longer than the lifetime of the cell. It is thus likely that the cell will not undergo a transition toward division. However, in the presence of large cyclin/Cdk overexpression (Figure 4C), the time needed for the switch to occur is much shorter.

Temporal evolution of Let-7 and cyclin B/Cdk1 indicates that the switch to cell proliferation triggered by cyclin overexpression is irreversible because down-regulation of cyclins to their initial levels will not restore cell cycle arrest (Supplementary Figure S3A, condition 1, 1200 h < *t* < 1500 h). This is the consequence of the irreversible bistable switch at the core of the transformation GRN (Gérard et al., 2014). The model predicts that a stronger decrease in cyclin synthesis can eventually stop cell proliferation, characterized by low, stable steady-state levels of cyclin B/Cdk1 (Supplementary Figure S3A, condition 3, *t* > 1500 h). The corresponding temporal evolution of the expression levels of Lin28 and STAT3, two critical activators of the transformation GRN, is shown in Supplementary Figure S3B. The model indicates that these cells (condition 3 for *t* > 1500 h) might be invasive cells, defined by high levels of STAT3 and Lin28 and low Let-7 levels, which are in a quiescent state (low levels of cyclin/Cdk).

Moreover, the model predicts that a transient down-regulation of Lin28 can impede both cell proliferation and the transformation network (Supplementary Figure S3C, condition 3, *t* > 1500 h), where low, stable levels of cyclin B/Cdk1 are present with high Let-7 levels. The corresponding temporal evolution of Lin28 and STAT3 expression levels is represented in Supplementary Figure S3D. Here also, modeling predictions agreed well with the experimental observations showing down-regulation of both cell proliferation and cell transformation after transient inhibition of Lin28 (Iliopoulos et al., 2009). To assess if the dynamical behavior of the model is robust against parameters variation, we perform sensitivity analysis. This analysis indicates that the rates of synthesis of the cyclin/Cdk complexes and the parameters driving the synthesis and degradation of Let-7 and LIN28 are important to control the dynamics of the cell cycle and cell transformation networks (see Supplementary Information and Supplementary Figure S4).

### GFs May Also Couple the Transformation and Cell Cycling Networks

After binding to specific ligands, tyrosine kinase receptors can lead to the activation of Ras (Schlessinger, 2000). Thus, in cells that possess active tyrosine kinase pathways, we expect that growth factors simultaneously activate cell proliferation, Ras, and the transformation network. The resulting coupling mechanisms between the proliferation and the transformation networks should thus be reinforced. We simulated the coupling mechanisms of the two networks *via* active tyrosine kinase signaling (Supplementary Figure S5). The model indicates that, even in the absence of Src (the triggering signal of the transformation network), GF is able to activate both the proliferation and the transformation networks. The model predicts that the network activation is faster for high levels of GF (compare Supplementary Figure S5B with Supplementary Figure S5C). Depending on cell types, GF may lead to the activation of cell proliferation through numerous different signaling pathways that do not necessarily depend on Ras. In the latter cells, we may thus suppose that GFs activate cell proliferation without affecting the transformation network *via* Ras. In the following, we do not take into account GF-mediated Ras-activation.

### Cancer-Type-Specific Activation of the Proliferation and Transformation Networks

The coupling of the two networks raised the question of their potential combined involvement in cancer. To address this issue, we first defined a cell proliferation index (*CPI*) and a non-transformed state index (*NTSI*), as follows

*CPI* = max(*Md*) + max(*Me*) + max(*Ma*) + max(*Mb*)

and

$$\begin{array}{c} NTSI=\frac{PTEN+Let7}{IL6+NFKB+STAT3+miR21+Lin28+Ras} \end{array}$$

*CPI* is the sum of the maximal RNA levels of all cyclin/Cdk complexes, which is, as a first approximation, an indication of cell proliferation; *NTSI* is the ratio between the RNA expression levels of the inhibitors, i.e., Let-7 and PTEN, divided by the expression levels of the activators of the epigenetic transformation switch, i.e., IL6, NF-κB, STAT3, miR21, Lin28, and Ras. Such ratio characterizes the degree of cell transformation where a high value defines a non-transformed cell while a low value corresponds to transformed cells. We here make use of the available gene expression data (TCGA) to measure CPI and NTSI in various cancers.

In addition, because the cell cycle is driven by the activity of successive Cdks and since mRNA and protein expression levels are not necessarily positively correlated, we collected, when available, the protein expression levels of the network components from the Cancer Proteome Atlas^1^. We showed that the mRNA and protein expression levels of CYCLIN B1, a major component driving mitosis entry, are positively correlated in the two studied cohorts, namely, hepatocellular carcinoma (HCC) and prostate adenocarcinoma (Supplementary Figures S6A,B). This already supports the choice of CPI, which is based on mRNA levels, as an indicator of cell proliferation.

To further support our indexes, we performed a principal component analysis (PCA) based on the mRNA expression levels of 124 components belonging to the cell cycle network pathway as defined in the Kyoto Encyclopedia of Genes and Genomes (KEGG)^2^ (Supplementary Figure S6C). This PCA performed on the HCC cohort (TCGA) permits the selection of 100 HCC samples with high cell cycle activation (red dots in Supplementary Figure S6C) and the other HCC samples defined as low cell cycle activation (gray dots). Indeed, HCCs with high cell cycle activation are located at the farthest position as compared to non-tumor samples (blue dots). CPIs are higher in HCC with high cell cycle activation compared to HCC with low cell cycle activation (Supplementary Figure S6D), which validates CPI as a good proxy of cell proliferation.

In addition, CPI measured in 20 HCC cell lines (RNASeq data from the OASIS Genomics portal)^3^ is much higher than CPI in HCC, which fits with the large cell proliferation capability of immortalized cell lines as compared to tumor samples (Supplementary Figure S6D).

Furthermore, in this work, the expression levels of Let-7 and cyclin B/Cdk1 were repeatedly used as markers of cell transformation and cell proliferation states (Figure 3, 4). As a support of this, we verified that in liver cancer, the expression of Let-7 and CYCLIN B1 mRNA is negatively correlated, in a similar manner, compared to NSTI *versus* CPI (Supplementary Figures S6E,F).

In conclusion, these analyses support the choice of CPI and NTSI as good indicators of cell proliferation and cell transformation states.

**FIGURE 5:**
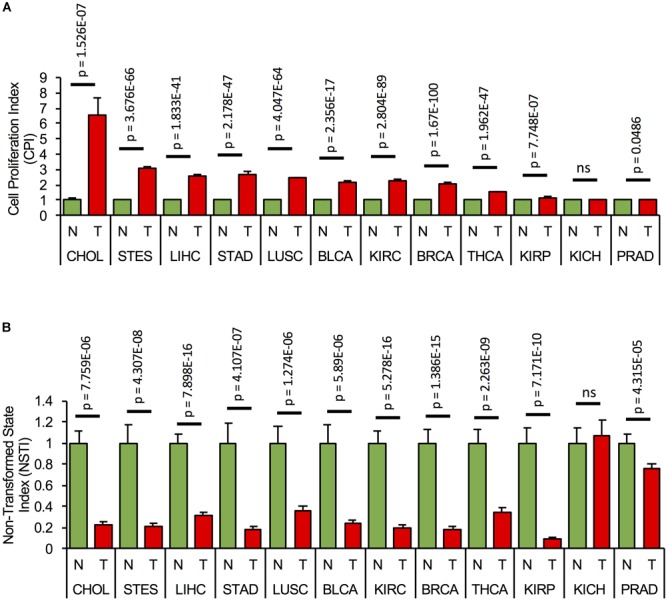
Concomitant activation of the proliferation and transformation networks is cancer type-specific. **(A)** Cell proliferation index, *CPI*, and **(B)** Non-transformed state index, *NSTI*, calculated from the measured mRNA expression levels of the network components in the non-tumor, normal (N) samples, (green bars) and tumor condition (T, red bars) of 12 tumor cohorts from TCGA. In each case, mRNA levels are relative to the non-tumor condition. A list of all components used in the analysis can be found in Supplementary Table S7.

Since the components of the cell cycling and transformation networks are expressed in several tissues, we assessed if the networks are activated, i.e., if their components are consistently misexpressed in different types of cancer. We first calculated *CPI* and *NSTI* based on the RNA expression of the network components (Supplementary Table S7) from the non-tumor (NT) and tumor samples (T) of TCGA cohorts of cholangiocarcinoma (CHOL: NT = 9, T = 36), stomach and esophageal carcinoma (STES: NT = 46, T = 600), HCC (LIHC: NT = 50, T = 369), stomach adenocarcinoma (STAD: NT = 35, T = 415), lung squamous cell carcinoma (LUSC: NT = 51, T = 501), bladder urothelial carcinoma (BLCA: NT = 19, T = 408), kidney renal clear cell carcinoma (KIRC: NT = 72, T = 534), breast carcinoma (BRCA: NT = 112, T = 1100), thyroid carcinoma (THCA: NT = 57, T = 510), kidney renal papillary cell carcinoma (KIRP: NT = 32, T = 290), kidney chromophobe (KICH: NT = 25, T = 66), and prostate adenocarcinoma (PRAD: NT = 52, T = 498) (Figure 5A,B). Significant and consistently high *CPI* and low *NSTI* values, as compared to non-tumor conditions, were obtained in several cancer types, with largest variations in gastrointestinal cancers, i.e., cholangiocarcinoma, hepatocellular, and stomach and esophageal carcinoma. Interestingly, the proliferation and transformation networks do not seem to be activated in kidney and prostate adenocarcinoma.

This suggests that concomitant activation of the cell proliferation and transformation networks is cancer-type-specific and predominantly occurs in gastrointestinal cancers.

### Patient-to-Patient Heterogeneity in the Activation of the Proliferation and Transformation Networks

**FIGURE 6:**
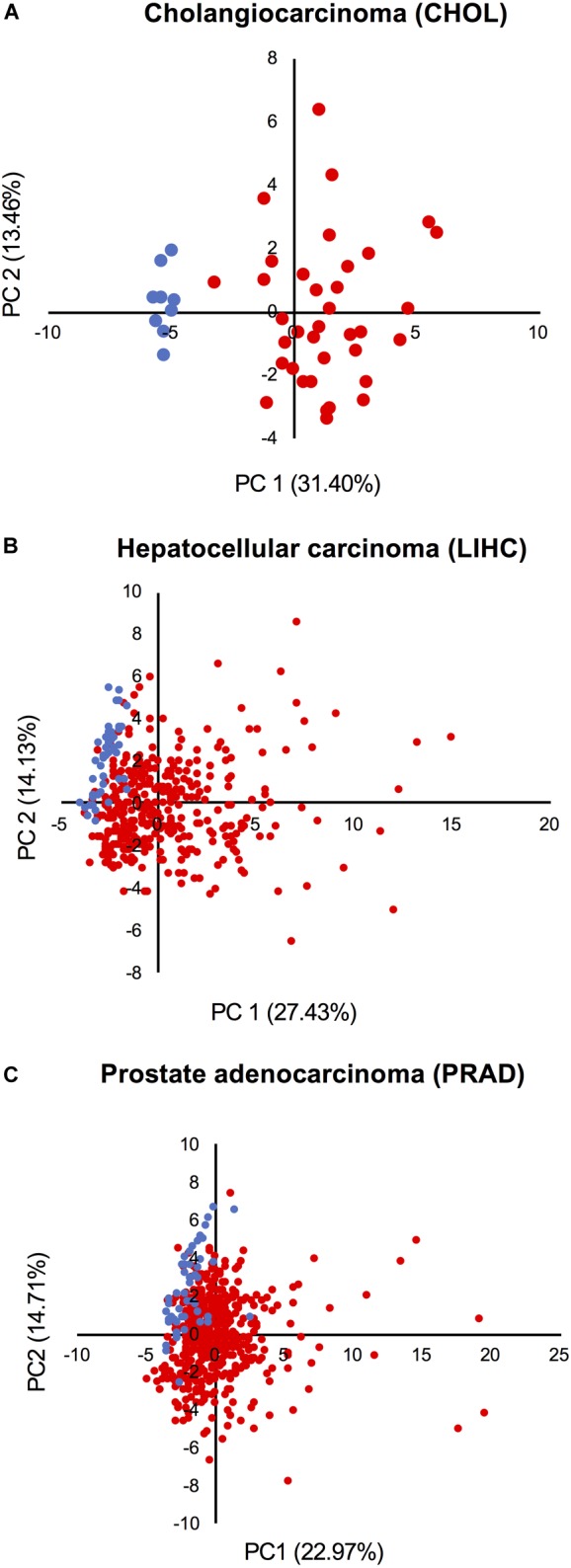
Activation of the networks in tumors is heterogeneous among patients. PCA based on the expression of all network components in the non-tumor (blue dots) and tumor condition (red dots) of **(A)** cholangiocarcinoma, **(B)** hepatocellular carcinoma, and **(C)** prostate adenocarcinoma cohort from TCGA.

Principal component analysis based on the expression of all network components was performed in three cohorts from TCGA: (1) the cholangiocarcinoma cohort, which displays the highest *CPI* levels; (2) the HCC cohort, which shows high *CPI* and low *NSTI* values; and (3) the prostate adenocarcinoma cohort characterized by low *CPI* and high *NSTI*. This analysis revealed that non-tumor (blue dots) and tumor samples (red dots) cluster separately in cholangiocarcinoma (Figure 6A), suggesting that the combined expression of the components of both networks can be used as a proxy to determine the tumorigenic state of a sample in this cohort.

A larger level of heterogeneity was detected within the HCC and prostate adenocarcinoma cohorts (Figure 6B,C). Moreover, some tumors cluster together with normal samples, indicating that the networks are not activated in all samples. Also, despite the fact that the mean *CPI* and *NSTI* values of prostate adenocarcinoma do not significantly differ from those in corresponding non-tumor samples (Figure 5A,B), some individual tumors are characterized by high activation of the networks (Figure 6C).

We concluded that the network activation varies from patient to patient and depends on tumor type.

### Dynamics of Cell Proliferation and Cell Transformation of a Heterogeneous Cell Population

**FIGURE 7:**
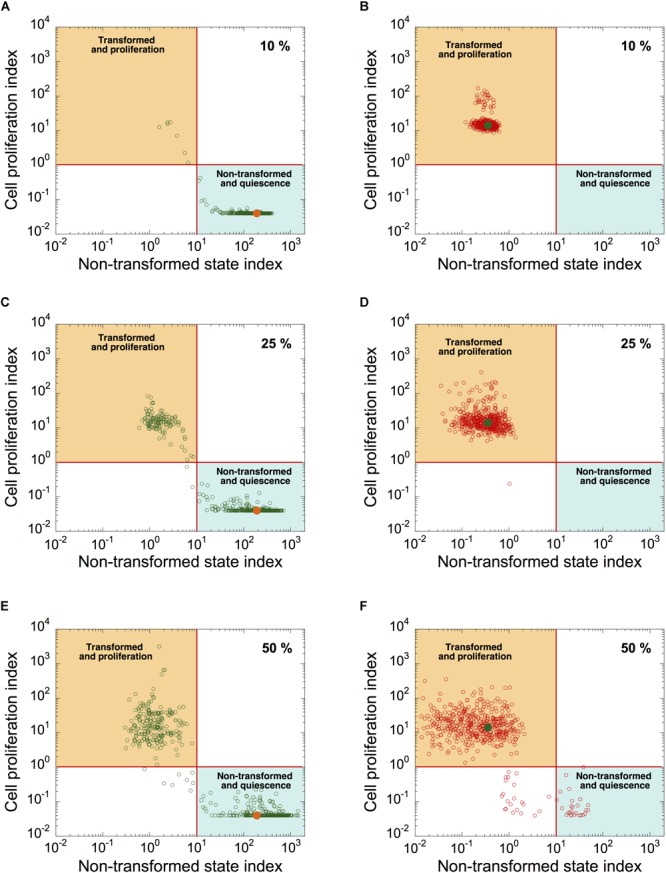
Modeling malignant cell transformation and cell proliferation distributions in a heterogeneous cell population. *CPI versus NSTI* is shown in a heterogeneous cell population starting from a non-transformed, quiescent, cell state **(A,C,E)** or from a transformed, proliferative, cell state **(B,D,F)**. In both states, 10% **(A,B)**, 25% **(C,D)**, or 50% **(E,F)** of uniform random variations from the basal value of each parameter are considered. Each circle corresponds to one cell in a population of 500 cells. Horizontal lines define an arbitrary threshold value for *CPI*, which is equal to 1, above which cells are considered into proliferation; vertical lines define an arbitrary threshold value for *NSTI*, equal to 10, above which cells are considered in a non-transformed state. Orange **(A,C,E)** and green dots **(B,D,F)** correspond to the value of both indexes in the absence of random variation on parameters. Initial conditions are given in Supplementary Table S6. Basal parameter values are as in Supplementary Table S5.

To determine the source of heterogeneity observed in patient samples, we incorporated a stochastic source of heterogeneity in a cell population model by applying, for each cell, uniform random variations around the basal value of each kinetic parameter. We plotted the simulated levels of *CPI* as a function of *NTSI* in a heterogeneous cell population of quiescent, non-transformed cells (Figure 7A,C,E; orange dots correspond to *CPI* and *NTSI* in the absence of random variations on parameter’s values), and in a population of transformed, proliferative cells (Figure 7B,D,F; green dots correspond to *CPI* and *NTSI* in the absence of random variations). Ten percent, 25%, and 50% of uniform random variations on each parameter were considered.

Simulations indicate that the non-transformed, quiescent cell state is less robust to random fluctuations than the transformed, proliferative state (compare Figure 7C with Figure 7D, and Figure 7E with Figure 7F). Indeed, starting in a non-transformed, quiescent cell state, a large proportion of cells switch to a transformed, proliferative state in the presence of random fluctuations in kinetic parameters (Figure 7C,E). However, from a transformed, proliferative cell state, only a small proportion of cells switch to a quiescent and non-transformed state (Figure 7F).

Thus, random fluctuations in kinetic parameter values could trigger abrupt switches in the dynamics of the cell cycling and transformation networks. The “non-tumor” state (quiescent, non-transformed cells) is more sensitive to random fluctuations in kinetic parameter values than “tumor” state (transformed, proliferative state).

We next assessed if the levels of key network components, i.e., Let-7, PTEN, or Ras, may affect the robustness of these cell states. In a heterogeneous population of non-transformed, quiescent cells with 25% of random parameter variations (Supplementary Figures S7A–F), an increase in Let-7 strengthens the robustness of the non-transformed, quiescent cell state toward random fluctuations in kinetic parameters and prevents random switches to a transformed, proliferative cell state (compare basal conditions in Supplementary Figure S7A with Supplementary Figures S7B–D where *V*_SLET7_ changes from 10 to 12, 20, and 50, respectively). Similarly, an increase in PTEN or a decrease in Ras also improved the robustness of the non-transformed, quiescent cell state (compare Supplementary Figure S7A with Supplementary Figures S7E,F).

Along the same lines, tumor suppressors and oncogenes can also impact the robustness of the transformed, proliferative cell state (Supplementary Figures S7G–L). Some cells revert to a non-transformed, quiescent state following a large increase in Let-7 (compare Supplementary Figure S7G with Supplementary Figures S7H–J). However, an increase in PTEN or a decrease in Ras (similar to the conditions in Supplementary Figures S7E,F) are unable to revert cells to a non-transformed, quiescent state (compare Supplementary Figure S7G with Supplementary Figures S7K,L).

Thus, an increase in tumor suppressors or a decrease in oncogenes reduces the probability of stochastic switches to a transformed, proliferative cell state. However, if cells are already in a proliferative, transformed state, similar changes in tumor suppressors or oncogenes do not permit reverting back to a more “healthy” cell phenotype, which highlights an irreversible process in cancer progression.

**FIGURE 8:**
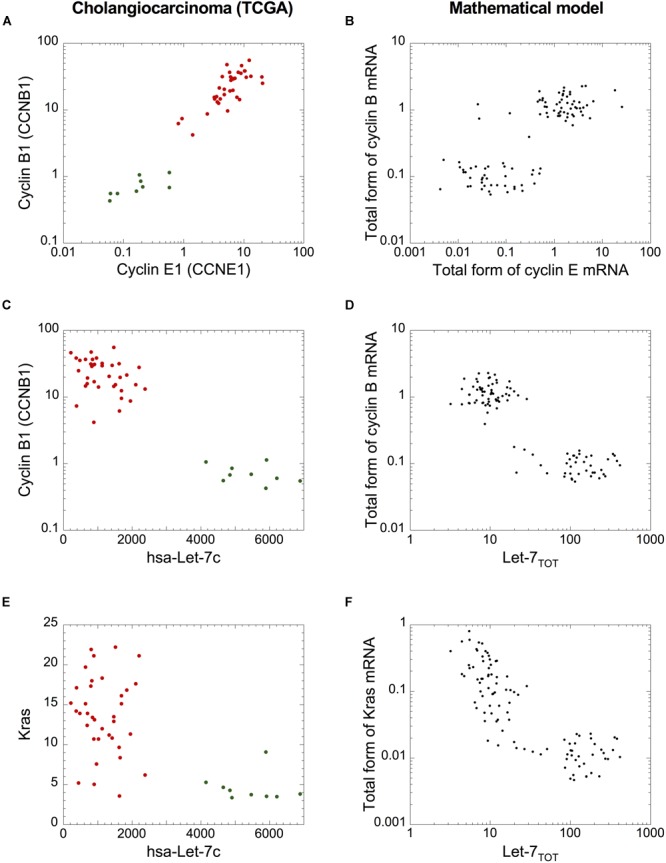
A cell population model accounts for the qualitative dynamics of the network switches in cholangiocarcinoma. mRNA levels of **(A,B)** cyclin B1 *versus* cyclin E1 **(C,D)**, cyclin B1 *versus* Let-7c, and **(E,F)** Kras *versus* Let-7c of cholangiocarcinoma cohort from TCGA **(A,C,E)** and **(B,D,F)** in a model for a heterogeneous cell population where 50% of uniform random variations are considered around the basal value of each parameter. **(B,D,F)** Simulations are performed with a population of 100 cells. Initial conditions are given in Supplementary Table S6. Src = 0.0000001 and other parameter values are as in Supplementary Table S5. **(A,C,E)** Green dots: non-tumor samples (*n* = 9), red dots: tumor samples (*n* = 36).

Finally, since cholangiocarcinomas are characterized by strong network activation (Figure 5, 6A), we analyzed if the cell population model can qualitatively reproduce the networks’ switch from normal to tumor condition. Plotting cyclin B1 mRNA as a function of cyclin E1 or Let-7c RNA (Figure 8A–D) and plotting Kras mRNA (representative as Ras) as a function of Let-7c (Figure 8E,F) revealed expression patterns that are qualitatively very similar to those predicted by the mathematical model of a heterogeneous cell population (Figure 8B,D,E). We concluded that the cell population model can be used to assess the stochastic dynamics of the switch of both networks in cholangiocarcinomas.

## Discussion

Tumorigenesis rests on many biological features, which include sustained proliferative signaling, evading growth suppressors, resisting cell death, promoting angiogenesis, ensuring replicative immortality, and eliciting invasion and metastasis (Hanahan and Weinberg, 2011). Here, we built a mathematical model to analyze the dynamical properties of a Let-7-dependent mechanism coupling cell proliferation and an epigenetic switch driving malignant transformation.

Our mathematical model illustrates qualitatively how Cdk-dependent and transformation networks may interact, and proposes a mechanism, acting through Let-7, which suggests that cyclin overexpression can promote cell proliferation, while inducing and accelerating malignant transformation. Indeed, overexpression of cyclins progressively sponges the free form of Let-7. The latter will no longer be available to repress the components of the transformation network, leading to the activation of the epigenetic switch. This effect is known as competing-endogenous, ceRNA, effect. CeRNAs regulate other RNA transcripts by competing for shared miRNA and were involved in tumorigenesis (Salmena et al., 2011; Tay et al., 2014; Chiu et al., 2017). Here, Let-7 is the shared miRNA between both networks. Let-7 was shown to be involved in different ceRNA mechanisms. Indeed, Let-7e can modulate the inflammatory response in vascular endothelial cells through a ceRNA effect (Lin et al., 2017). Imprinted H19 lncRNA, which plays important roles in development, cancer, and metabolism, modulates Let-7 availability by acting as a molecular sponge and causing precocious muscle differentiation (Kallen et al., 2013). Moreover, amplification of MYCN mRNA levels in neuroblastoma can sponge Let-7, thereby rendering LIN28B dispensable for cancer progression (Powers et al., 2016). Note, however, that experimental and theoretical studies indicate that a ceRNA effect between multiple RNA transcripts and the shared miRNA is effective only in the presence of adequate expression levels of the transcripts and the miRNA (Gérard and Novak, 2013; Denzler et al., 2014; Tay et al., 2014).

Our mathematical model further shows that stochastic variations in kinetic parameter values from cell to cell can create large fluctuations in the global network dynamics, possibly leading to stochastic switches of some cells to a transformed and proliferative state. To determine to which extent tumor heterogeneity could be explained by cell-to-cell stochastic switches in the network dynamics, cohorts of single-cell transcriptomic data of tumors would be useful. Stochastic switches in the GRN dynamics could indeed be a source of heterogeneity in cancer cell populations (Tang, 2012; Patel et al., 2014). Stochastic switches in gene networks were identified in hematopoietic tumor stem cells (Dingli et al., 2007), in the appearance of mammary tumor in mice (Bouchard et al., 1989), and in the differentiation and maturation of T lymphocytes (Davis et al., 1993). These switches in single-cell behaviors may also promote phenotypic equilibrium in population of cancer cells (Gupta et al., 2011). Transcriptomic analysis of TCGA data suggests that the coupling between the cell cycle and malignant transformation networks and the activation of these networks in tumors are cancer-type-specific, with predominant activation in gastrointestinal cancers. Our PCA reveals inter-patient heterogeneity in network activation in tumors, which stresses the need to consider patient-specific characteristics when optimizing therapeutic strategies aiming to reverse the network dynamics of activated GRNs (Biankin et al., 2015). Inter-patient heterogeneity in the activation of two networks might be the consequence of different tumor developmental stages in each patient. In addition, since each tumor is characterized by intratumoral heterogeneity, the location of the tumor biopsy within each patient may also generate heterogeneity in the network activation.

In conclusion, by means of transcriptomic data analysis and modeling-based investigations, we identified a Let-7-dependent connection between two major GRNs involved in tumorigenesis, and whose activation is cancer- and patient-specific. In the future, it will be interesting to incorporate in the model the additional regulatory links between the transformation network and the cell cycle (Coqueret and Gascan, 2000; Bienvenu et al., 2001; Brantley et al., 2001; Ding et al., 2008; Xiong et al., 2008; Xu et al., 2009) and to examine their contribution in the dynamics resulting from the coupling between the two networks and in particular in the propensity of the cell to switch into a proliferative, potentially cancerous, state. We anticipate that a better characterization of the dynamics resulting from the combination of other GRNs specific for each patient will help provide a global GRN activation map for personalizing and optimizing cancer treatment.

## Author Contributions

All authors designed the study, conceived the model, analyzed the results, and read, revised, and approved the manuscript. CG and DG wrote the codes for the numerical simulations. CG performed the numerical simulations and wrote the manuscript.

## Conflict of Interest Statement

The authors declare that the research was conducted in the absence of any commercial or financial relationships that could be construed as a potential conflict of interest.

## Footnotes

1 https://tcpaportal.org/tcpa/index.html

2 https://www.genome.jp/kegg/pathway.html

3 http://www.oasis-genomics.org/
