# Supplementary material for: Modeling the Dynamics of Let-7-Coupled Gene Regulatory Networks Linking Cell Proliferation to Malignant Transformation
Source: Front Physiol. 2019 Jul 11;10:848. doi: 10.3389/fphys.2019.00848 (PMC6637753; doi:10.3389/fphys.2019.00848)
Supplement: Supplementary file 1 [file Data_Sheet_1.pdf]

Supplementary Information

Modeling the dynamics of Let-7-coupled gene regulatory networks linking cell proliferation to malignant transformation

Claude Gérard[1], Frédéric Lemaigre[1]and Didier Gonze[2]
[1]de Duve Institute, Université catholique de Louvain, Brussels, Belgium
[2]Unité de Chronobiologie théorique, Faculté des Sciences,
Université libre de Bruxelles, Brussels, Belgium

Table of contents

Description of the mathematical model 2

Numerical simulations and PCA analysis 2

Analysis of RNASeq and proteomic data 2

Sensitivity analysis of the cell cycle and cell transformation networks 3

Supplementary tables 4

- **Table S1. Variables of the mathematical model** 4
- **Table S2. Predicted *CYCLIN*, *CDK* and *E2F* mRNAs targets of human Let-7 miRNA** .. 5
- **Table S3. Kinetic equations of the mathematical model** 6
- **Table S4. Detailed kinetic reactions of the mathematical model** 9
- **Table S5. Parameters of the model** 12
- **Table S6. Initial conditions** 15
- **Table S7. List of the components used in the human tumors analysis (Figs. 5-6)** 16

Supplementary figures 17

- **Figure S1. Detailed scheme of the model** 17
- **Figure S2. Cell proliferation vs cell cycle arrest mediated by Let-7 and GF levels** 18
- **Figure S3. Effect of the Cdk network on the transformation network dynamics** 19
- **Figure S4. Sensitivity analysis** 20
- **Figure S5. Growth factors couple transformation and proliferation networks in a Let-7-independent manner.** 21
- **Figure S6. CPI correlates with cell cycle activation** 22
- **Figure S7. Oncogenes and tumor suppressors control the robustness of the networks dynamics** 23

Supplementary references 24

Description of the mathematical model

The model is composed of set of 15 kinetic equations governing the time dynamics of the Cdk network and 14 kinetics equations governing the dynamics of the inflammatory circuit which controls malignant transformation. The different variables of the model are defined in Table S1, the kinetics equations are found in Table S2, while the description of the parameters and their numerical values used in the simulations are in Table S5.

In the model, we consider for simplicity a constant total concentration of NF-κB ($NFKB_{TOT}$), E2F ($E2F_{TOT}$), and APC (APC_{TOT}). Activation and inactivation reactions of E2F in Eq. [5], APC in Eq. [15] and NF-κB in Eq. [16] behave as Goldbeter-Koshland switches (Goldbeter and Koshland, 1981). All other processes of the model rest on mass-action kinetics. Moreover, to couple the model for the Cdk network with the model for the inflammatory circuit leading to malignant transformation, equations [1] and [18] have been replaced by equation [18'].

Numerical simulations and PCA analysis

Numerical simulations were performed with XPPAUTO (http://www.math.pitt.edu/~bard/xpp/xpp.html) and matlab. The model is available as xpp file on GitHub (https://github.com/dgonze/Cell-cycle), and as SBML on BioModels (https://www.ebi.ac.uk/biomodels/MODEL1906070001).

Pearson correlation analysis were performed with rcorr function (Hmisc package), while PCA analysis were done with FactoMineR (Le, 2008).

Analysis of RNASeq and proteomic data

RNASeq and miRNASeq data of the different patient cohorts were retrieved from TCGA database (http://firebrowse.org/). For each cohort, we converted the

“scaled_estimate” in the “illuminahiseq_rnaseqv2_unc_edu_Level_3_RSEM_genes” file into TPM by multiplying by 10^6.

Proteomic data from different cohorts in TCGA originate from The Cancer Proteome Atlas (https://tcpaportal.org/tcpa/index.html), where we used the RPPA dataset with the level 4 of data normalization.

RNASeq data of the 20 HCC cell lines arise from the OASIS Genomics portal (http://www.oasis-genomics.org/).

Sensitivity analysis of the cell cycle and cell transformation networks

Qualitative dynamics of the GRN, such as sustained oscillatory regime, stable steady states and bistable switches, critically depend on the GRN structure and the presence of gene regulatory motifs, i.e. positive and/or negative feedback loops (Tyson et al., 2003), but they may be also sensitive to kinetic parameter values. To assess the robustness of the behaviour with respect to parameter values, we performed a sensitivity analysis to identify which parameters of the mathematical model mostly influence the network dynamics. We increased or decreased by 20% the value of each parameter of the cell cycle network (Fig. S4A, B) and of the cell transformation network (Fig. S4C). For the cell cycle network, we plotted the change in amplitude and the change in period of the sustained oscillations of Cyclin B/Cdk1. The analysis indicated that the rates of synthesis of the cyclin/Cdk complexes, especially cyclin B/Cdk1, cyclin E/Cdk2 and cyclin A/Cdk2 are important parameters to control the amplitude and the period of the cyclin/Cdk oscillations. For the cell transformation network, we reported the change in the stable steady state level of Let-7 and we noted that the parameters driving the synthesis and degradation of Let-7 and LIN28 are important to control the levels of Let-7, and may induce a switch in the network if they are altered.

Supplementary tables

Table S1. Variables of the mathematical model

Name	Definition
Model for the Cdk network, regulated by Let-7, driving the mammalian cell cycle	
Let7	MicroRNA family Let-7
mMd	Cyclin D mRNA
Md	Active form of cyclin D/Cdk-4-6 complex
mMdlet7	Inactive complex between cyclin D mRNA (mMd) and Let7
E2F	Active form of the transcription factor E2F
mMe	Cyclin E mRNA
Me	Active form of cyclin E/Cdk2 complex
mMelet7	Inactive complex between cyclin E mRNA (mMe) and Let7
mMa	Cyclin A mRNA
Ma	Active form of cyclin A/Cdk2 complex
mMalet7	Inactive complex between cyclin A mRNA (mMa) and Let7
mMb	Cyclin B mRNA
Mb	Active form of cyclin B/Cdk1 complex
mMblet7	Inactive complex between cyclin B mRNA (mba) and Let7
APC	Anaphase-promoting complex regulating cyclin degradation
Model for the inflammatory circuit driving the epigenetic switch to cell transformation	
Let7	MicroRNA family Let-7
NFKB	Transcription factor NF-κB
Lin28	RNA binding protein Lin28
mIL6	IL6 mRNA
mIL6Let7	Complex form between mIL6 and Let7
IL6	Interleukin 6 protein, which promotes the epigenetic switch leading to cell transformation
mRas	Ras oncogene mRNA
mRasLet7	Complex form between mRas and Let7
Ras	Protein form of Ras oncogene
STAT3	Transcription factor STAT3, whose activity is crucial to promote cell transformation
miR21	MicroRNA miR21, which represses the translation of the tumor suppressor PTEN
mPTEN	PTEN mRNA
miRmpten	Complex form between miR21 and mPTEN
PTEN	Protein form of the tumor suppressor PTEN (phosphatase and tensin homologue)

Table S2. Predicted *CYCLIN*, *CDK* and *E2F* mRNAs targets of human Let-7 miRNA

miRNA	mRNA targets with their (score)*
hsa-let-7a-2-3p	**CCNB1** (70), E2F5 (54), E2F3 (79)
hsa-let-7a-3p	**CCNA2** (98), **CCNE2** (92), **CDK6** (71), E2F5 (90), E2F6 (73), E2F7 (76), E2F8 (92)
hsa-let-7a-5p	**CCND1** (65), **CCND2** (98), E2F2 (83), E2F5 (94), E2F6 (91)
hsa-let-7b-3p	**CDK6** (71), **CCNA2** (98), **CCNE2** (92), E2F8 (92), E2F5 (90), E2F7 (76), E2F6 (73)
hsa-let-7b-5p	E2F5 (94), E2F6 (91), E2F2 (83), **CCND2** (98), **CCND1** (65)
hsa-let-7c-3p	**CCNB2** (72), E2F4 (92), E2F3 (91)
hsa-let-7c-5p	E2F2 (83), E2F5 (94), E2F6 (91), **CCND1** (65), **CCND2** (98)
hsa-let-7d-5p	E2F5 (94), E2F6 (90), E2F2 (83), **CCND1** (56), **CCND2** (98)
hsa-let-7e-5p	E2F5 (94), E2F6 (91), E2F2 (83), **CCND2** (98), **CCND1** (65)
hsa-let-7f-1-3p	**CCNA2** (98), **CCNE2** (92), **CDK6** (71), E2F8 (92), E2F5 (90), E2F7 (76), E2F6 (73)
hsa-let-7f-2-3p	E2F6 (92), E2F5 (55), E2F8 (51)
hsa-let-7f-5p	**CCND1** (65), **CCND2** (98), E2F5 (94), E2F6 (91), E2F2 (83)

*The analysis was performed with miRDB, an online database for miRNA target prediction (http://mirdb.org/). Note that E2F family members can also be targeted by Let-7. In the mathematical model, to avoid an increase of the number of kinetic equations, we do not explicitly consider these regulations. Since E2F is a transcription factor that promote cyclin synthesis, a Let-7-mediated reduction of E2F protein level will reduce the synthesis of the different cyclins. Dynamically, this will be similar to a direct inhibition of the production of cyclin/Cdk complexes though binding of the cyclin to Let-7.

Table S3. Kinetic equations of the mathematical model

Model for the Cdk network, regulated by Let-7, driving the mammalian cell cycle	
$\frac{dLet7}{dt} = V_{\mathrm{SLET7}} - k_1 \cdot mMd \cdot Let7 + k_2 \cdot mMdLet7 - k_3 \cdot mMe \cdot Let7 + k_4 \cdot mMeLet7$ $-k_5 \cdot mMa \cdot Let7 + k_6 \cdot mMaLet7 - k_7 \cdot mMb \cdot Let7 + k_8 \cdot mMbLet7 - k_{\mathrm{DLET7}} \cdot Let7$	[1]
$\frac{dmMd}{dt} = V_{SMCYC} \cdot k_{SMMD} \cdot GF - k_1 \cdot mMd \cdot Let7 + k_2 \cdot mMdLet7 - k_{DMMD} \cdot mMd$	[2]
$\frac{dMd}{dt} = k_{\mathrm{SD}} \cdot mMd - k_{\mathrm{DMD}} \cdot Md$	[3]
$\frac{dmMdLet7}{dt} = k_1 \cdot mMd \cdot Let7 - k_2 \cdot mMdLet7 - k_{\mathrm{DMMDLET7}} \cdot mMdLet7$	[4]
$\frac{dE2F}{dt} = k_{\mathrm{1E2F}} \cdot \left(\frac{(E2F_{\mathrm{TOT}} - E2F)}{K_{\mathrm{AE2F}} + (E2F_{\mathrm{TOT}} - E2F)} \right) \cdot (Md + Me) - k_{\mathrm{2E2F}} \cdot \left(\frac{E2F}{K_{\mathrm{IE2F}} + E2F} \right) \cdot Ma$	[5]
$\frac{dmMe}{dt} = V_{\mathrm{SMCYC}} \cdot k_{\mathrm{SMME}} \cdot E2F - k_3 \cdot mMe \cdot Let7 + k_4 \cdot mMeLet7 - k_{\mathrm{DMME}} \cdot mMe$	[6]
$\frac{dMe}{dt} = k_{\mathrm{SE}} \cdot mMe - k_{\mathrm{DME}} \cdot Ma \cdot \left(\frac{Me}{K_{\mathrm{DE}} + Me} \right) - k_{\mathrm{DME2}} \cdot Me$	[7]
$\frac{dmMeLet7}{dt} = k_3 \cdot mMe \cdot Let7 - k_4 \cdot mMeLet7 - k_{\mathrm{DMMELET7}} \cdot mMeLet7$	[8]
$\frac{dmMa}{dt} = V_{\mathrm{SMCYC}} \cdot k_{\mathrm{SMMA}} \cdot E2F - k_5 \cdot mMa \cdot Let7 + k_6 \cdot mMaLet7 - k_{\mathrm{DMMA}} \cdot mMa$	[9]
$\frac{dMa}{dt} = k_{\mathrm{SA}} \cdot mMa - k_{\mathrm{DMA}} \cdot APC \cdot \left(\frac{Ma}{K_{\mathrm{DA}} + Ma} \right) - k_{\mathrm{DMA2}} \cdot Ma$	[10]
$\frac{dmMaLet7}{dt} = k_5 \cdot mMa \cdot Let7 - k_6 \cdot mMaLet7 - k_{\mathrm{DMMALET7}} \cdot mMaLet7$	[11]
$\frac{dmMb}{dt} = V_{\mathrm{SMCYC}} \cdot V_{\mathrm{SMMB}} - k_7 \cdot mMb \cdot Let7 + k_8 \cdot mMbLet7 - k_{\mathrm{DMMB}} \cdot mMb$	[12]
$\frac{dMb}{dt} = k_{SB} \cdot mMb \cdot Ma - k_{DMB} \cdot APC \cdot \frac{Mb}{K_{DB} + Mb} - k_{DMB2} \cdot Mb$	[13]

$$\frac{dmMbLet7}{dt} = k_7 \cdot mMb \cdot Let7 - k_8 \cdot mMbLet7 - k_{\mathrm{DMMBLET7}} \cdot mMbLet7 \qquad [14]$$

$$\frac{dAPC}{dt} = k_{1APC} \cdot \left(\frac{APC_{TOT} - APC}{K_{AAPC} + (APC_{TOT} - APC)}\right) \cdot Mb - V_{2APC} \cdot \left(\frac{APC}{K_{1APC} + APC}\right) \qquad [15]$$

Model for the inflammatory circuit driving the epigenetic switch to cell transformation

$$\frac{dNFKB}{dt} = \left(k_{\mathrm{AA1NFKB}} \cdot Src + k_{\mathrm{AA2NFKB}} \cdot IL6 + k_{\mathrm{AA3NFKB}} \cdot Ras\right) \cdot \left(\frac{K_{\mathrm{IPTEN}}}{K_{\mathrm{IPTEN}} + PTEN}\right) \cdot \left(\frac{NFKB_{\mathrm{I}}}{K_{\mathrm{ANFKB}} + NFKB_{\mathrm{I}}}\right)$$
$$-V_{\mathrm{DNFKB}} \cdot \left(\frac{NFKB}{K_{\mathrm{INFKB}} + NFKB}\right) \qquad [16]$$

$$\frac{dLIN28}{dt} = V_{\mathrm{SLIN28}} \cdot \left(\frac{NFKB}{K_{\mathrm{A1NFKB}} + NFKB}\right) - k_{\mathrm{DLIN28}} \cdot LIN28 \qquad [17]$$

$$\frac{dLet7}{dt} = V_{\mathrm{SLET7}} \cdot \left(\frac{K_{\mathrm{ILET7}}}{K_{\mathrm{ILET7}} + LIN28}\right) - k_9 \cdot mIL6 \cdot Let7 + k_{10} \cdot mIL6Let7 - k_{11} \cdot mRas \cdot Let7 + k_{12} \cdot mRasLet7$$
$$-k_{\mathrm{DLET7}} \cdot Let7 \qquad [18]$$

$$\frac{dmIL6}{dt} = V_{\mathrm{S1MIL6}} + V_{\mathrm{S2MIL6}} \cdot \left(\frac{NFKB}{K_{\mathrm{A2NFKB}} + NFKB}\right) - k_9 \cdot mIL6 \cdot Let7 + k_{10} \cdot mIL6Let7 - k_{\mathrm{DMIL6}} \cdot mIL6 \qquad [19]$$

$$\frac{dmIL6Let7}{dt} = k_9 \cdot mIL6 \cdot Let7 - k_{10} \cdot mIL6Let7 - k_{\mathrm{DILLET}} \cdot mIL6Let7 \qquad [20]$$

$$\frac{dIL6}{dt} = k_{SIL6} \cdot mIL6 - k_{DIL6} \cdot IL6 \qquad [21]$$

$$\frac{dmRas}{dt} = V_{\mathrm{SMRAS}} - k_{11} \cdot mRas \cdot Let7 + k_{12} \cdot mRasLet7 - k_{\mathrm{DMRAS}} \cdot mRas \qquad [22]$$

$$\frac{dmRasLet7}{dt} = k_{11} \cdot mRas \cdot Let7 - k_{12} \cdot mRasLet7 - k_{\mathrm{DRASLET}} \cdot mRasLet7 \qquad [23]$$

$$\frac{dRas}{dt} = k_{SRAS} \cdot mRas - k_{DRAS} \cdot Ras \qquad [24]$$

$$\frac{dSTAT3}{dt} = V_{SSTAT} \cdot \left(\frac{IL6}{K_{A2IL6} + IL6}\right) - k_{DSTAT} \cdot STAT3 \qquad [25]$$

$$\frac{dmiR21}{dt} = V_{SMIR21} \cdot \left(\frac{STAT3}{K_{ASTAT} + STAT3}\right) - k_{13} \cdot miR21 \cdot mPTEN + k_{14} \cdot miRmPTEN - k_{DMIR21} \cdot miR21$$	[26]
$$\frac{dmPTEN}{dt} = V_{\mathrm{SMPTEN}} - k_{13} \cdot miR21 \cdot mPTEN + k_{14} \cdot miRmPTEN - k_{\mathrm{DMPTEN}} \cdot mPTEN$$	[27]
$$\frac{dPTEN}{dt} = k_{\mathrm{SPTEN}} \cdot mPTEN - k_{\mathrm{DPTEN}} \cdot PTEN$$	[28]
$$\frac{dmiRmPTEN}{dt} = k_{13} \cdot miR21 \cdot mPTEN - k_{14} \cdot miRmPTEN - k_{\mathrm{DMIRMP}} \cdot miRmPTEN$$	[29]
Coupling the Cdk network and the epigenetic switch through Let-7 miRNA	
$$\frac{dLet7}{dt} = V_{\mathrm{SLET7}} \cdot \left(\frac{K_{\mathrm{ILET7}}}{K_{\mathrm{ILET7}} + LIN28}\right) - k_9 \cdot mIL6 \cdot Let7 + k_{10} \cdot mIL6Let7 - k_{11} \cdot mRas \cdot Let7 + k_{12} \cdot mRasLet7$$ $$-k_1 \cdot mMd \cdot Let7 + k_2 \cdot mMdLet7 - k_3 \cdot mMe \cdot Let7 + k_4 \cdot mMeLet7 - k_5 \cdot mMa \cdot Let7 + k_6 \cdot mMaLet7$$ $$-k_7 \cdot mMb \cdot Let7 + k_8 \cdot mMbLet7 - k_{\mathrm{DLET7}} \cdot Let7$$	[18’]
Simulations where GF promote cyclin D synthesis and activate Ras	
$$\frac{dRas}{dt} = k_{S1RAS} \cdot mRas + k_{S2RAS} \cdot GF - k_{DRAS} \cdot Ras$$	[24’]

Where:

$$NFKB_{\mathrm{TOT}} = NFKB + NFKB_{\mathrm{I}}$$

Table S4. Detailed kinetic reactions of the mathematical model

Reaction number (Fig. S1)	Definition of the reaction	Reaction
1	Activation of NFKB by Src	$k_{\mathrm{AA1NFKB}} \cdot Src \cdot \left(\frac{K_{\mathrm{IPTEN}}}{K_{\mathrm{IPTEN}} + PTEN}\right) \cdot \left(\frac{NFKB_I}{K_{\mathrm{ANFKB}} + NFKB_I}\right)$
2	Activation of NFKB by Ras	$k_{\mathrm{AA3NFKB}} \cdot Ras \cdot \left(\frac{K_{\mathrm{IPTEN}}}{K_{\mathrm{IPTEN}} + PTEN}\right) \cdot \left(\frac{NFKB_I}{K_{\mathrm{ANFKB}} + NFKB_I}\right)$
3	Activation of LIN28 synthesis by NFKB	$V_{\mathrm{SLIN28}} \cdot \left(\frac{NFKB}{K_{\mathrm{A1NFKB}} + NFKB}\right)$
4	Inhibition of Let-7 by LIN28	$V_{\mathrm{SLET7}} \cdot \left(\frac{K_{\mathrm{ILET7}}}{K_{\mathrm{ILET7}} + LIN28}\right)$
5	Association/dissociation of Let-7 and mIL6 (inhibition of mIL6 translation by Let-7)	$-\, k_9 \cdot \mathrm{mIL6} \cdot \mathrm{Let7} + k_{10} \cdot \mathrm{mIL6Let7}$
6	Association/dissociation of Let-7 and mRas (inhibition of mRas translation by Let-7)	$-\, k_{11} \cdot \mathrm{mRas} \cdot \mathrm{Let7} + k_{12} \cdot \mathrm{mRasLet7}$
7	Activation of NFKB by IL6	$k_{\mathrm{AA2NFKB}} \cdot IL6 \cdot \left(\frac{K_{\mathrm{IPTEN}}}{K_{\mathrm{IPTEN}} + PTEN}\right) \cdot \left(\frac{NFKB_I}{K_{\mathrm{ANFKB}} + NFKB_I}\right)$
8	Activation of mIL6 transcription by NFKB	$V_{\mathrm{S2MIL6}} \cdot \left(\frac{NFKB}{K_{\mathrm{A2NFKB}} + NFKB}\right)$
9	Activation of STAT3 synthesis by IL6	$k_{\mathrm{SSTAT}} \cdot \left(\frac{IL6}{K_{\mathrm{A2IL6}} + IL6}\right)$
10	Activation of miR-21 synthesis by STAT3	$k_{\mathrm{SMIR21}} \cdot \left(\frac{STAT3}{K_{\mathrm{ASTAT}} + STAT3}\right)$
11	Association/dissociation of miR-21 and mPTEN (inhibition of mPTEN translation by miR-21)	$-\, k_{13} \cdot \mathrm{miR21} \cdot \mathrm{mPTEN} + k_{14} \cdot \mathrm{miRmPTEN}$
12	Inhibition of NFKB by PTEN	See reactions 1, 2 and 7
13	Association/dissociation of Let-7 and mMd (inhibition of mMd translation by Let-7)	$-\, k_1 \cdot \mathrm{mMd} \cdot \mathrm{Let7} + k_2 \cdot \mathrm{mMdLet7}$
14	Association/dissociation of Let-7 and mMe (inhibition of mMe translation by Let-7)	$-\, k_3 \cdot \mathrm{mMe} \cdot \mathrm{Let7} + k_4 \cdot \mathrm{mMeLet7}$
15	Association/dissociation of Let-7 and mMa (inhibition of mMa translation by Let-7)	$-\, k_5 \cdot \mathrm{mMa} \cdot \mathrm{Let7} + k_6 \cdot \mathrm{mMaLet7}$
16	Association/dissociation of Let-7 and mMb (inhibition of mMb translation by Let-7)	$-\, k_7 \cdot \mathrm{mMb} \cdot \mathrm{Let7} + k_8 \cdot \mathrm{mMbLet7}$
17	Activation of mMd transcription by GF	$V_{\mathrm{SMYC}} \cdot k_{\mathrm{SMMD}} \cdot GF$
18	Transcription rate of mMd	See reaction 17
18	Translation of Md	$k_{\mathrm{SD}} \cdot \mathrm{mMd}$
19	Degradation of mMd	$-\, k_{\mathrm{DMMD}} \cdot mMd$
19	Degradation of Md	$-\, k_{\mathrm{DMD}} \cdot \mathrm{Md}$
20	Activation of E2F by Md	$k_{\mathrm{1E2F}} \cdot \left(\frac{E2F_{TOT} - E2F}{K_{\mathrm{AE2F}} + E2F_{TOT} - E2F}\right) \cdot Md$
21	Activation of E2F by Me	$k_{\mathrm{1E2F}} \cdot \left(\frac{E2F_{TOT} - E2F}{K_{\mathrm{AE2F}} + E2F_{TOT} - E2F}\right) \cdot Me$
22	Activation of mMe transcription by E2F	$V_{\mathrm{SMYC}} \cdot k_{\mathrm{SMME}} \cdot \mathrm{E2F}$
22	Translation of Me	$k_{\mathrm{SE}} \cdot \mathrm{mMe}$

23	Degradation of mMe	$- k_{DMME} \cdot mMe$
23	Degradation of Me	$- k_{DME2} \cdot Me$
24	Activation of Me degradation by Ma	$k_{DME} \cdot Ma \cdot \left(\frac{Me}{K_{DE} + Me}\right)$
25	Activation of mMe transcription by E2F	See Reaction 22
26	Activation of mMa transcription by E2F	$V_{SMYC} \cdot k_{SMMA} \cdot E2F$
27	Inactivation of E2F by Ma	$- k_{2E2F} \cdot \left(\frac{E2F}{K_{IE2F} + E2F}\right) \cdot Ma$
28	Activation of E2F	See Reactions 20 and 21
29	Inactivation of E2F	See Reaction 27
30	mMa transcription	See Reaction 26
30	Ma translation	$k_{SA} \cdot mMa$
31	Degradation of mMa	$- k_{DMMA} \cdot mMa$
32	Degradation of Ma	$- k_{DMA2} \cdot Ma$
33	Transcription of mMb	$V_{SMCYC} \cdot V_{SMMB}$
33	Activation of Mb synthesis by Ma	$k_{SB} \cdot mMb \cdot Ma$
34	Degradation of mMb	$- k_{DMMB} \cdot mMb$
34	Degradation of Mb	$- k_{DMB2} \cdot Mb$
35	Activation of APC promoted by Mb	$k_{1APC} \cdot \left(\frac{APC_{TOT} - APC}{K_{AAPC} + APC_{TOT} - APC}\right) \cdot Mb$
36	Activation of APC	See Reaction 35
37	Inactivation of APC	$V_{2APC} \cdot \left(\frac{APC}{K_{IAPC} + APC}\right)$
38	Degradation of Mb activated by APC	$- k_{DMB} \cdot APC \cdot \left(\frac{Mb}{K_{DB} + Mb}\right)$
39	Degradation of Ma activated by APC	$- k_{DMA} \cdot APC \cdot \left(\frac{Ma}{K_{DA} + Ma}\right)$
	**For sake of simplicity, the following reactions were not numbered in the wiring diagram of Fig. S1**	
	Synthesis rate of Let7	V_{SLET7}
	Degradation rate of Let7	$- k_{DLET7} \cdot Let7$
	Activation of LIN28 synthesis by NFKB	$V_{SLIN28} \cdot \left(\frac{NFKB}{K_{A1NFKB} + NFKB}\right)$
	Degradation of mMdLet7	$- k_{DMMDLET7} \cdot mMdLet7$
	Degradation of mMeLet7	$- k_{DMMELET7} \cdot mMeLet7$
	Degradation of mMaLet7	$- k_{DMMALET7} \cdot mMaLet7$
	Degradation of mMbLet7	$- k_{DMMBLET7} \cdot mMbLet7$
	Inactivation of NFKB	$- V_{DNFKB} \cdot \left(\frac{NFKB}{K_{INFKB} + NFKB}\right)$
	Degradation of LIN28	$- k_{DLIN28} \cdot LIN28$
	Basal transcription rate of mIL6	V_{S1MIL6}
	Transcription of mIL6 activated by NFKB	$V_{S2MIL6} \cdot \left(\frac{NFKB}{K_{A2NFKB} + NFKB}\right)$
	Degradation of mIL6	$- k_{DMIL6} \cdot mIL6$
	Degradation of mIL6Let7	$- k_{DILLET} \cdot mIL6Let7$
	Translation of IL6	$k_{SIL6} \cdot mIL6$

	Degradation of IL6	$-k_{DIL6} \cdot IL6$
	Transcription of mRas	V_{SMRAS}
	Degradation of mRas	$-k_{DMRAS} \cdot mRas$
	Degradation of mRasLet7	$-k_{DRASLET} \cdot mRasLet7$
	Translation of Ras	$k_{SRAS} \cdot mRas$
	Degradation of Ras	$-k_{DRAS} \cdot Ras$
	Synthesis of STAT3 activated by IL6	$V_{SSTAT} \cdot \left(\frac{IL6}{K_{A2IL6} + IL6}\right)$
	Degradation of STAT3	$-k_{DSTAT} \cdot STAT3$
	Synthesis of miR21 activated by STAT3	$V_{SMIR21} \cdot \left(\frac{STAT3}{K_{ASTAT} + STAT3}\right)$
	Degradation of miR21	$-k_{DMIR21} \cdot miR21$
	Transcription of mPTEN	V_{SMPTEN}
	Degradation of mPTEN	$-k_{DMPTEN} \cdot mPTEN$
	Translation of PTEN	$k_{SPTEN} \cdot mPTEN$
	Degradation of PTEN	$-k_{DPTEN} \cdot PTEN$
	Degradation of miRmPTEN	$-k_{DMIRMP} \cdot miRmPTEN$

Table S5. Parameters of the model

Symbol	Definition	Numerical value
**Model for the Cdk network, regulated by Let-7, driving the mammalian cell cycle**		
V_{SMCYC}	Multiplying factor for the rates of synthesis of all cyclins	1.5
GF	Growth factors	1
k_{SMMD}	Transcription rate constant of cyclin D	0.03
k_{DMMD}	Rate constant for the degradation of cyclin D messenger RNA	0.2
k_{SD}	Translation rate constant of cyclin D	0.5
k_{DMD}	Rate constant for the degradation of cyclin D protein	0.5
k_{1E2F}	Rate constant for activation of E2F	2.3
K_{AE2F}	Michaelis constant for E2F activation	0.01
k_{2E2F}	Rate constant for inactivation of E2F	2.5
K_{IE2F}	Michaelis constant for E2F inactivation	0.01
$E2F_{TOT}$	Total level of E2F transcription factor	4
k_{SMME}	Transcription rate constant of cyclin E	0.035
k_{DMME}	Rate constant for the degradation of cyclin E messenger RNA	0.08
k_{SE}	Translation rate constant of cyclin E	0.8
k_{DME}	Rate constant for the degradation of cyclin E protein regulated by cyclin A/Cdk2	0.2
k_{DME2}	Basal rate constant for the degradation of cyclin E protein	0.1
K_{DE}	Michaelis constant for degradation of cyclin E/Cdk2 by cyclin A/Cdk2	0.08
k_{SMMA}	Transcription rate constant of cyclin A	0.07
k_{DMMA}	Rate constant for the degradation of cyclin A messenger RNA	0.08
k_{SA}	Translation rate constant of cyclin A	0.4
k_{DMA}	Rate constant for the degradation of cyclin A protein regulated by APC	0.35
k_{DMA2}	Basal rate constant for the degradation of cyclin A protein	0.05
K_{DA}	Michaelis constant for degradation of cyclin A/Cdk2 by APC	0.01
V_{SMMB}	Transcription rate of cyclin B	0.06
k_{DMMB}	Rate constant for the degradation of cyclin B messenger RNA	0.08
k_{SB}	Translation rate constant of cyclin B	0.35
k_{DMB}	Rate constant for the degradation of cyclin B protein regulated by APC	0.2
k_{DMB2}	Basal rate constant for the degradation of cyclin B protein	0.7
K_{DB}	Michaelis constant for degradation of cyclin B/Cdk1 by APC	0.01
k_{1APC}	Rate constant for activation of APC through cyclin B/Cdk1	1
K_{AAPC}	Michaelis constant for APC activation	2
V_{2APC}	Inactivation rate of APC	0.3
K_{IAPC}	Michaelis constant for APC inactivation	10
APC_{TOT}	Total level of the Anaphase-Promoting Complex	2
k_1	Bimolecular rate constant for binding of Let7 to mMd	1
k_2	Rate constant for dissociation of complex (mMdlet7) between Let7 and mMd	0.01
k_3	Bimolecular rate constant for binding of Let7 to mMe	1
k_4	Rate constant for dissociation of complex (mMelet7) between Let7 and	0.01

	mMe	
k_5	Bimolecular rate constant for binding of Let7 to mMa	1
k_6	Rate constant for dissociation of complex (mMalet7) between Let7 and mMa	0.01
k_7	Bimolecular rate constant for binding of Let7 to mMb	1
k_8	Rate constant for dissociation of complex (mMblet7) between Let7 and mMb	0.01
$k_{DMMDLET7}$	Rate constant for degradation of mMdlet7 complex	1
$k_{DMMELET7}$	Rate constant for degradation of mMelet7 complex	1
$k_{DMMALET7}$	Rate constant for degradation of mMalet7 complex	1
$k_{DMMBLET7}$	Rate constant for degradation of mMblet7 complex	1
Model for the inflammatory circuit driving the epigenetic switch to cell transformation		
Src	Src kinase oncoprotein (signal triggering an inflammatory response leading to an activation of the epigenetic switch)	0
V_{SLET7}	Maximum rate of synthesis of Let7 microRNA	10
K_{ILET7}	Michaelis constant for the repression of Let7 synthesis by Lin28	0.1
k_{DLET7}	Rate constant for degradation of Let7 microRNA	0.05
$k_{AA1NFKB}$	Rate constant for the activation of NF-κB by Src	10
$k_{AA2NFKB}$	Rate constant for the activation of NF-κB by IL6	0.09
$k_{AA3NFKB}$	Rate constant for the activation of NF-κB by Ras	1
K_{ANFKB}	Michaelis constant for the activation of NF-κB	0.01
K_{INFKB}	Michaelis constant for the inhibition of NF-κB	0.02
V_{DNFKB}	Maximum rate for NF-κB inactivation	0.01
$NFKB_{TOT}$	Total concentration of NF-κB	1
K_{A2IL6}	Michaelis constant for the activation of STAT3 synthesis by IL6	40
K_{IPTEN}	Michaelis constant for the inhibition of NF-κB activation by PTEN	5
V_{SLIN28}	Maximum rate of synthesis of Lin28	0.1
k_{DLIN28}	Rate constant for the degradation of Lin28	0.015
K_{A1NFKB}	Michaelis constant for the activation of Lin28 synthesis by NF-κB	0.01
k_9	Bimolecular rate constant for binding of Let7 to mIL6	10
k_{10}	Rate constant for dissociation of complex (mIL6let7) between Let7 and mIL6	0.01
V_{S1MIL6}	Independent rate of synthesis of IL6 mRNA, mIL6	0.12
V_{S2MIL6}	Maximum rate of synthesis of IL6 mRNA depending on NF-κB	0.01
k_{DMIL6}	Rate constant for the degradation of IL6 mRNA	0.01
k_{DILLET}	Rate constant for the degradation of the complex between Let7 and mIL6	0.5
K_{A2NFKB}	Michaelis constant for the activation of mIL6 synthesis by NF-κB	5
k_{SIL6}	Rate constant for the synthesis of IL6 protein	1.5
k_{DIL6}	Rate constant for the degradation of IL6 protein	0.4
V_{SMRAS}	Rate of synthesis of Ras mRNA, mRas	0.006
k_{11}	Bimolecular rate constant for binding of Let7 to mRas	10
k_{12}	Rate constant for dissociation of complex (mRaslet7) between Let7 and mRas	0.01
k_{DMRAS}	Rate constant for the degradation of mRas	0.01
$k_{DRASLET}$	Rate constant for the degradation of the complex between Let7 and mRas	0.5
k_{SRAS}	Rate constant for the synthesis of Ras protein	0.25

k_{DRAS}	Rate constant for the degradation of Ras protein	0.03
V_{SSTAT}	Maximum rate of synthesis of STAT3	0.15
k_{DSTAT}	Rate constant for the degradation of STAT3	0.03
V_{SMIR21}	Maximum rate of synthesis of miR21 microRNA	4
K_{ASTAT}	Michaelis constant for the activation miR21 synthesis by STAT3	5
k_{13}	Bimolecular rate constant for binding of miR21 to mPTEN	10
k_{14}	Rate constant for dissociation of complex (miRmpten) between miR21 and mPTEN	0.01
k_{DMIR21}	Rate constant for the degradation of miR21	0.2
V_{SMPTEN}	Rate of synthesis of PTEN mRNA, mPTEN	0.001
k_{DMPTEN}	Rate constant for the degradation of mPTEN	0.1
k_{DMIRMP}	Rate constant for the degradation of the complex between mPTEN and miR21	0.1
k_{SPTEN}	Rate constant for the synthesis of PTEN protein	0.9
k_{DPTEN}	Rate constant for the degradation of PTEN protein	0.05

Note: We focus our study on qualitative rather than quantitative aspects in the expression of the different components of the network. Indeed, our goal is to analyze the dynamic implications of the regulatory structure of the epigenetic switch linking inflammation to cell transformation and of the Cdk network driving the cell cycle, i.e. the wiring diagram, or the topology, which is crucial for the network behavior (see (Wagner, 2005)). Moreover, many parameters have not been determined experimentally. The numerical values have been selected to yield a non-transformed state without the inflammatory signal, Src; or a transformed state within less than 100h with transient inflammatory signal, which corresponds to experimental observations (Iliopoulos et al., 2009). For the Cdk network, parameter values have been chosen to reach cell cycle periods ranging between 20h and 50h, which correspond to the doubling time in most mammalian cells (Alexiades and Cepko, 1996). The time units for the parameters in the model are expressed in hours, while the concentrations are expressed in μM. In addition, the mechanisms controlling miRNA degradation are complex and not fully understood (Zhang et al., 2012). As a consequence, we assume that each complex between a mRNA and its regulating miRNA is rapidly targeted for degradation. The rate constants for the degradation of mRNAs and proteins are between $0.01h^{-1}$ and $1h^{-1}$. This range of values corresponds to a half-life duration between 0.69h and 69.3h, which is in agreement with experimentally-measured half-life durations (Schwanhausser et al., 2011). Indeed, Schwanhäusser *et al.* showed that the mean half-life duration is 9h and 46h, for mRNAs and proteins, respectively. In the model, the short half-life durations of 0.69h are the half-life durations of the complexes between Let-7 and the mRNAs of the different cyclins. Such short half-life durations are in line with the fact that miRNA binding to its target mRNAs rapidly leads to its degradation (Wu et al., 2006).

Table S6. Initial conditions

Variable	C.I. # 1 (leads to non-transformed cell)	C.I. # 2 (leads to transformed cell)	C.I. # 3 (cell population, Figs. 8B,D,F)
NFKB	0.00045	0.5	0.01
Lin28	0.34	0.5	0.01
Let7	40	1	0
mIL6	0.0003	0.5	0.01
IL6	0.001	0.5	0.01
mRas	0.00001	0.5	0.01
Ras	0.0001	0.5	0.01
STAT3	0.0001	0.5	0.01
mPTEN	0.01	0	0.01
mRasLet7	0	0	0
mIL6Let7	0	0	0
miR21	0.0003	0	0
PTEN	0.17	0	0
MiRmPTEN	0	0	0

Table S7. List of the components used in the human tumors analysis (Figs. 5-6)

# of components	Symbol	Name
1	CCNA1	Cyclin A1
2	CCNB1	Cyclin B1
3	CCND1	Cyclin A1
4	CCNE1	Cyclin E1
5	CDK1	Cyclin-dependent kinase 1
6	CDK2	Cyclin-dependent kinase 2
7	CDK4	Cyclin-dependent kinase 4
8	CDK6	Cyclin-dependent kinase 6
9	E2F1	E2F transcription factor 1
10	E2F2	E2F transcription factor 2
11	E2F3	E2F transcription factor 3
12	E2F4	E2F transcription factor 4
13	E2F5	E2F transcription factor 5
14	E2F6	E2F transcription factor 6
15	E2F7	E2F transcription factor 7
16	E2F8	E2F transcription factor 8
17	IL6R	Interleukin 6 receptor
18	IL6	Interleukin 6
19	KRAS	KRAS oncogene
20	LIN28A	Lin-28 homolog A
21	LIN28B	Lin-28 homolog B
22	PTEN	Phosphatase and tensin homolog
23	STAT3	Signal transducer and activator of transcription 3
24	hsa-let-7a-1	MicroRNA let-7a-1
25	hsa-let-7a-2	MicroRNA let-7a-2
26	hsa-let-7a-3	MicroRNA let-7a-3
27	hsa-let-7b	MicroRNA let-7b
28	hsa-let-7c	MicroRNA let-7c
29	hsa-let-7d	MicroRNA let-7d
30	hsa-let-7e	MicroRNA let-7e
31	hsa-mir-21	MicroRNA 21

Supplementary figures

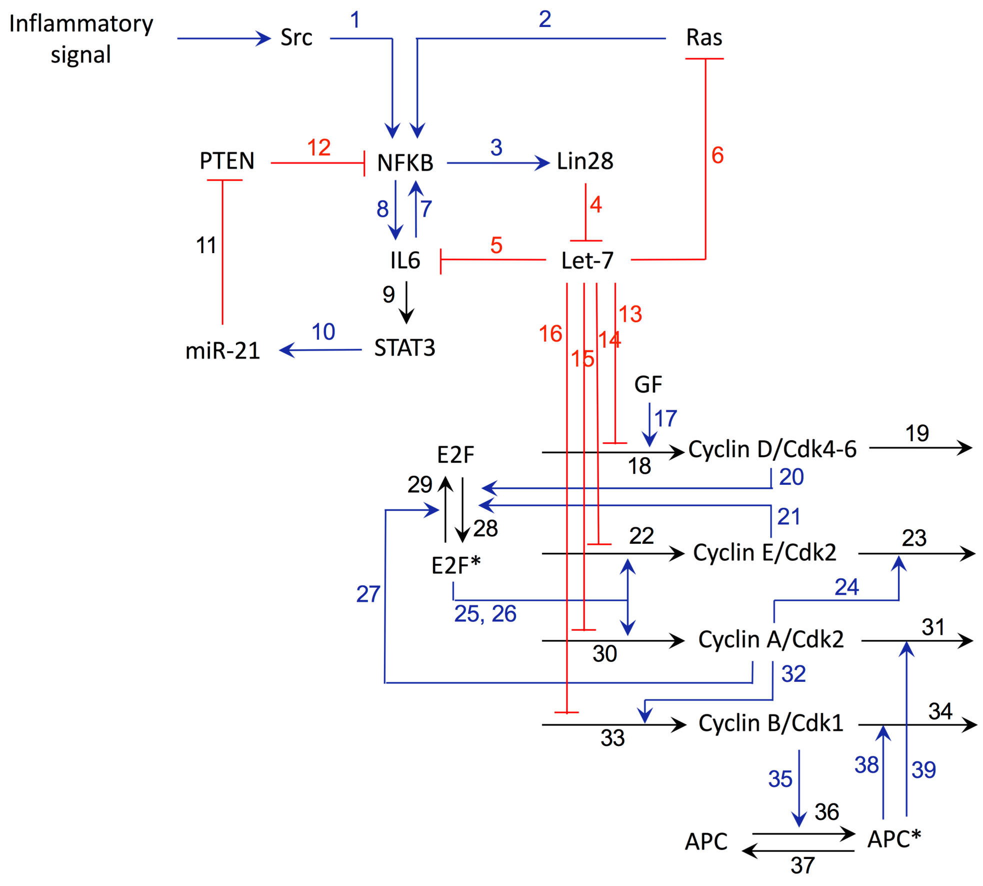

Figure S1. Detailed scheme of the model. The model for the epigenetic switch linking inflammation to cell transformation originates from a previous study (Gerard et al., 2014), while the model for the post-transcriptional regulation of the Cdk network by Let-7 is based on a skeleton model for the Cdk network driving the mammalian cell cycle (Gerard and Goldbeter, 2011). Let-7, which is involved in multiple positive feedback loops, can repress the synthesis of each cyclin. Numbers correspond to the equations given in Table S4.

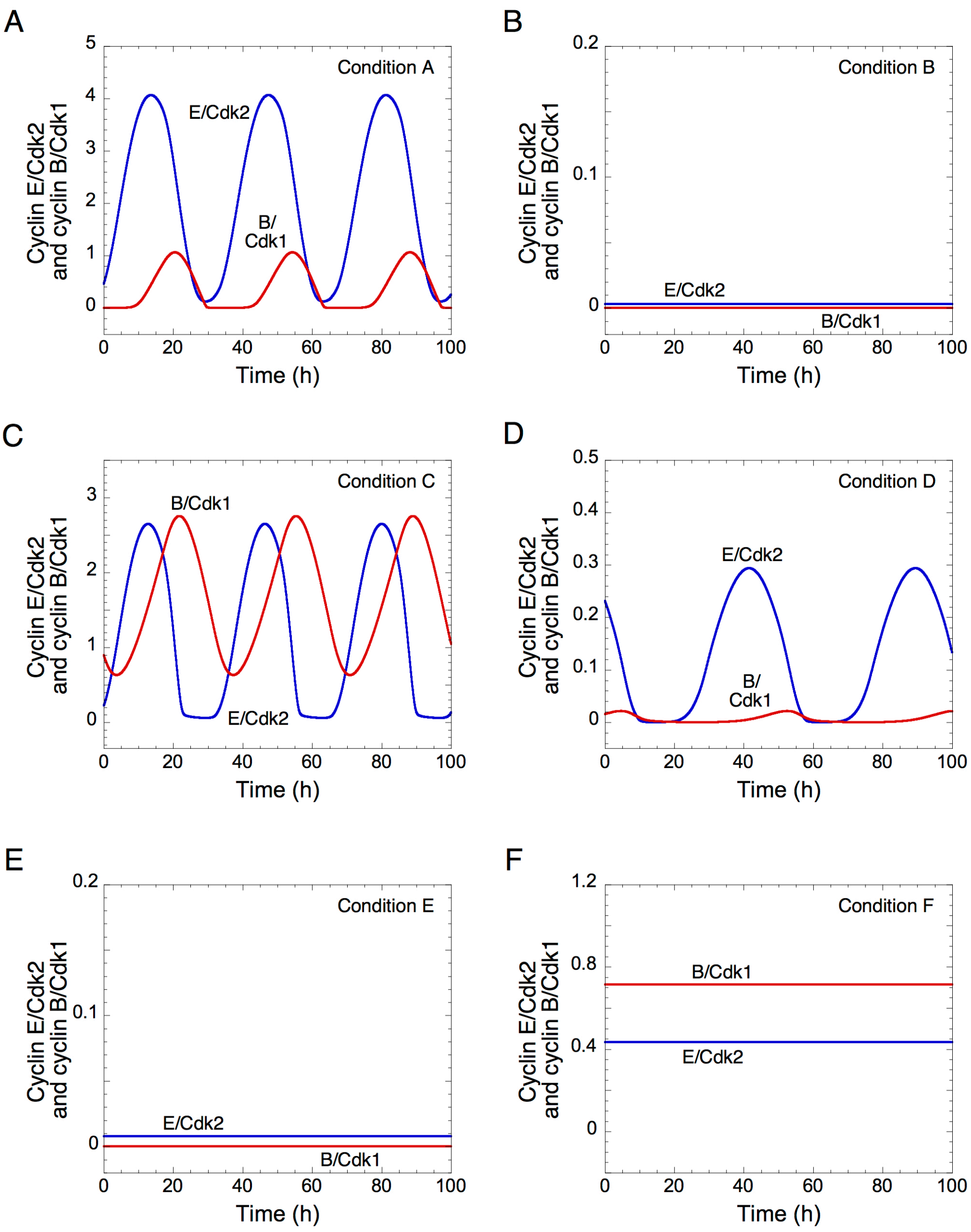

Figure S2. Cell proliferation vs cell cycle arrest mediated by Let-7 and GF levels. The temporal evolutions of cyclin E/Cdk2 (blue) and cyclin B/Cdk1 (red) in panels A to F correspond to conditions A to F of panel A from Fig. 2. In A, GF = 3 and V_{SLET7} = 0.25; in B, GF = 3 and V_{SLET7} = 2; in C, GF = 25 and V_{SLET7} = 0.25; in D, GF = 25 and V_{SLET7} = 1.6; in E, GF = 25 and V_{SLET7} = 5; and in F, GF = 70 and V_{SLET7} = 1.5. Other parameter values are as in Table S5.

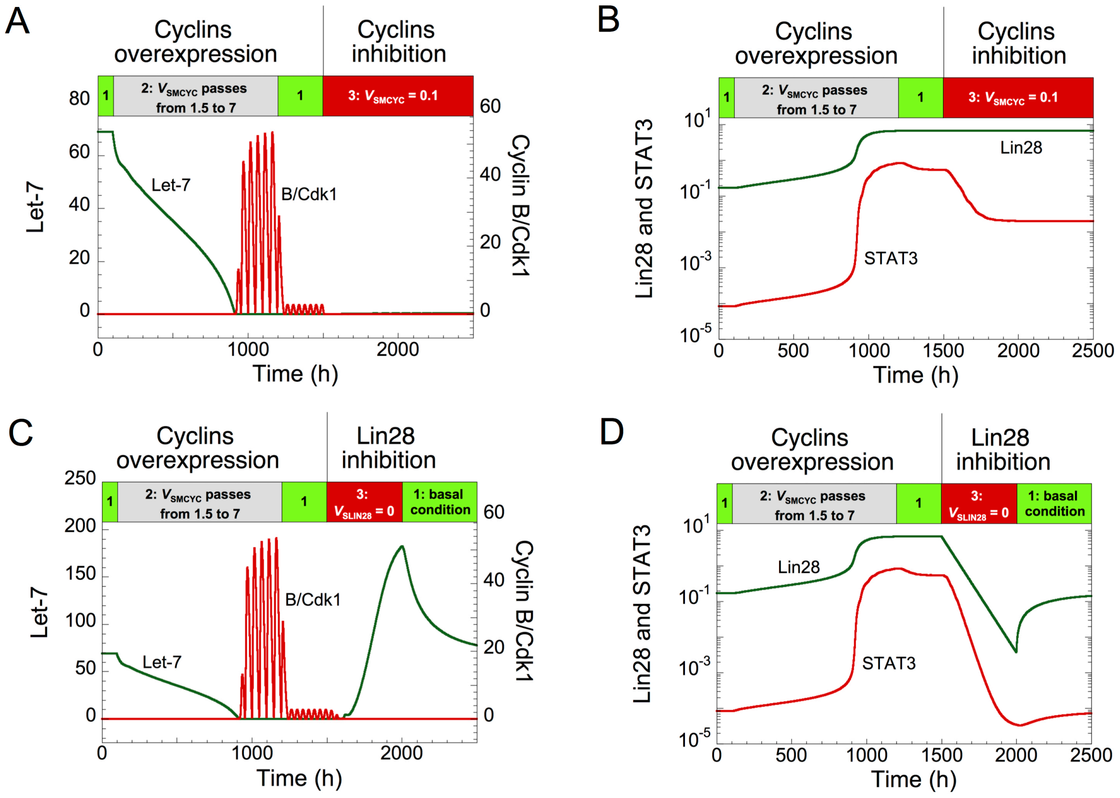

Figure S3. Effect of the Cdk network on the transformation network dynamics. (A, C) Temporal evolution of Let-7 and cyclin B/Cdk1. From a non-transformed and quiescent cell state, V_{SMCYC} changes from 1.5 to 7 (at t = 100h), and promotes the epigenetic switch and cell proliferation. For 1200 h < t < 1500 h, V_{SMCYC} is set back to its initial value (= 1.5), but cell proliferation and the transformed cell state are maintained, which is characterized by sustained oscillations in cyclin B/Cdk1 (with the same amplitude as in Fig. 1B) and low levels in Let-7. In A, at t = 1500h, V_{SMCYC} changes from 1.5 to 0.1, preventing cell proliferation without recovering a non-transformed cell state since cyclin B/Cdk1 tends to a low stable steady state level, while Let-7 levels remain low. In C, for 1500h < t < 2000h, the synthesis rate of Lin28, V_{SLIN28} is set to 0, allowing the recovery of a stable non-transformed state defined by high levels of Let-7 and impeding cell proliferation, characterized by a low, stable steady state, level of cyclin B/Cdk1. In C, for t > 2000h, basal conditions are used. (B, D) Temporal evolution of Lin28 and STAT3 corresponds to conditions of panels A and C, respectively. Other parameter values are as in Table S5, which correspond to basal conditions.

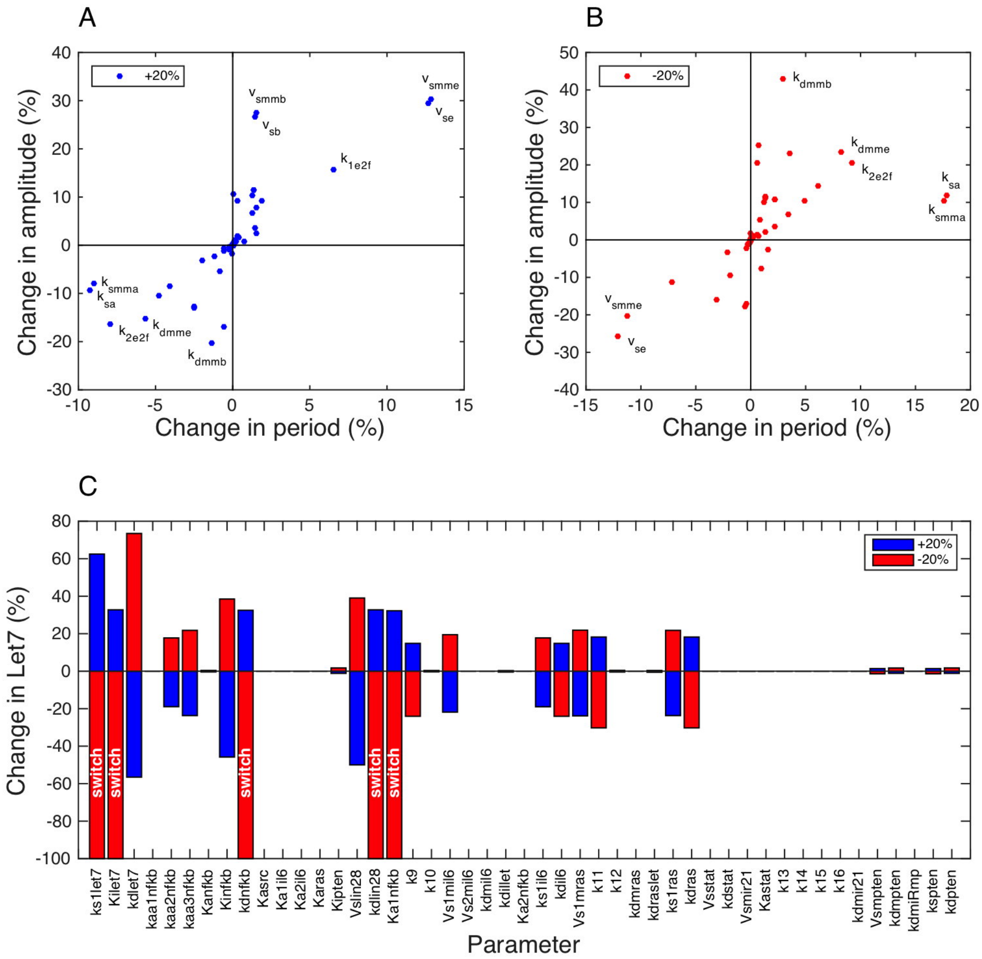

Figure S4. Sensitivity analysis. (A,B) Effect of changes in parameter values on the cell cycle. Each dot corresponds to an increase (A) or a decrease (B) of 20% of one parameter value. On the y-axis, 'amplitude' refers to the amplitude of Cyclin B/Cdk1 (variable Mb). The name of the parameters which influence the most the period and amplitude of the cell cycle are indicated. (C) Effect of changes in parameter values on the level of Let7 (in the condition where Let7 is activated and the cell cycle arrested). Blue/red bars refer respectively to an increase/decrease of 20% of one parameter value. Only parameters related to the transformation network are reported. Note that a decrease of some parameter values induces a switch of the transformation network (associated to the entry into a proliferative state). A change of 20% in the value of a single parameter of the cell cycle have virtually no effect of the level of Let7. As shown in Fig. S3, an over-expression of the cyclins may, however, activates the transformation network and trigger the cell cycle. Basal parameter values are as in Table S5.

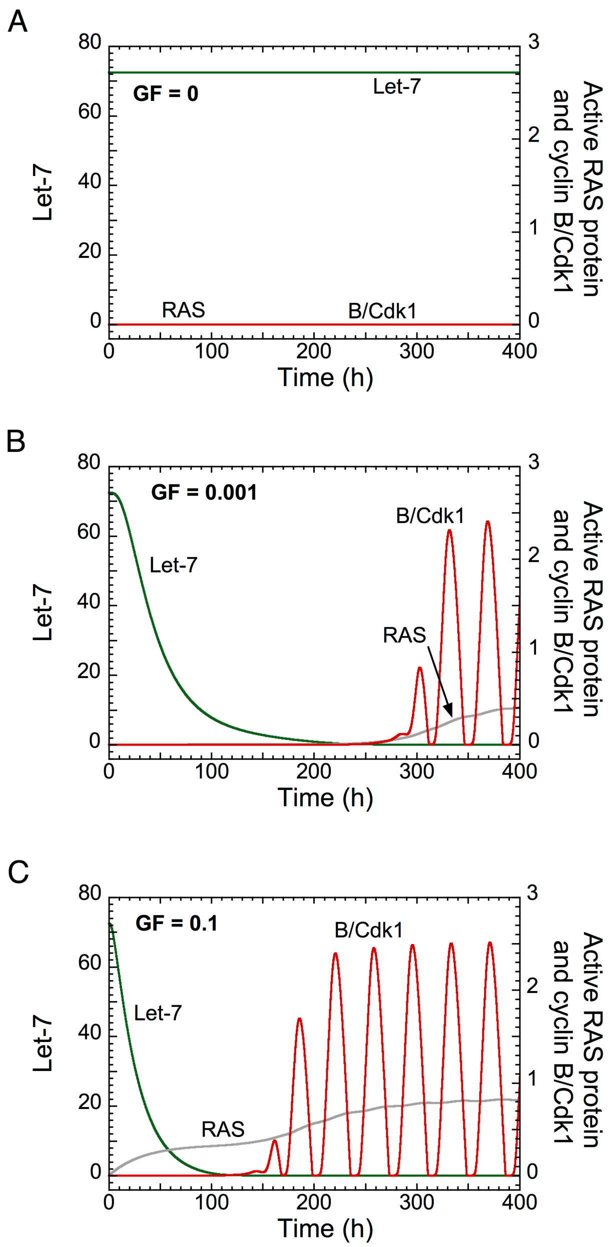

Figure S5. Growth factors couple transformation and proliferation networks in a Let-7-independent manner. Temporal evolution of Let-7 (green curve), the active form of RAS protein (grey curve) and cyclin B/Cdk1 (red curve) in the absence of GF (A), in the presence of low, GF = 0.001 in B, and higher levels of GF, GF = 0.1 in C. k_{S2RAS} in Eq. [24'] is equal to 0.1. Other parameter values and kinetic equations are as in Supplementary Information where Eq. [24] was replaced by Eq. [24'].

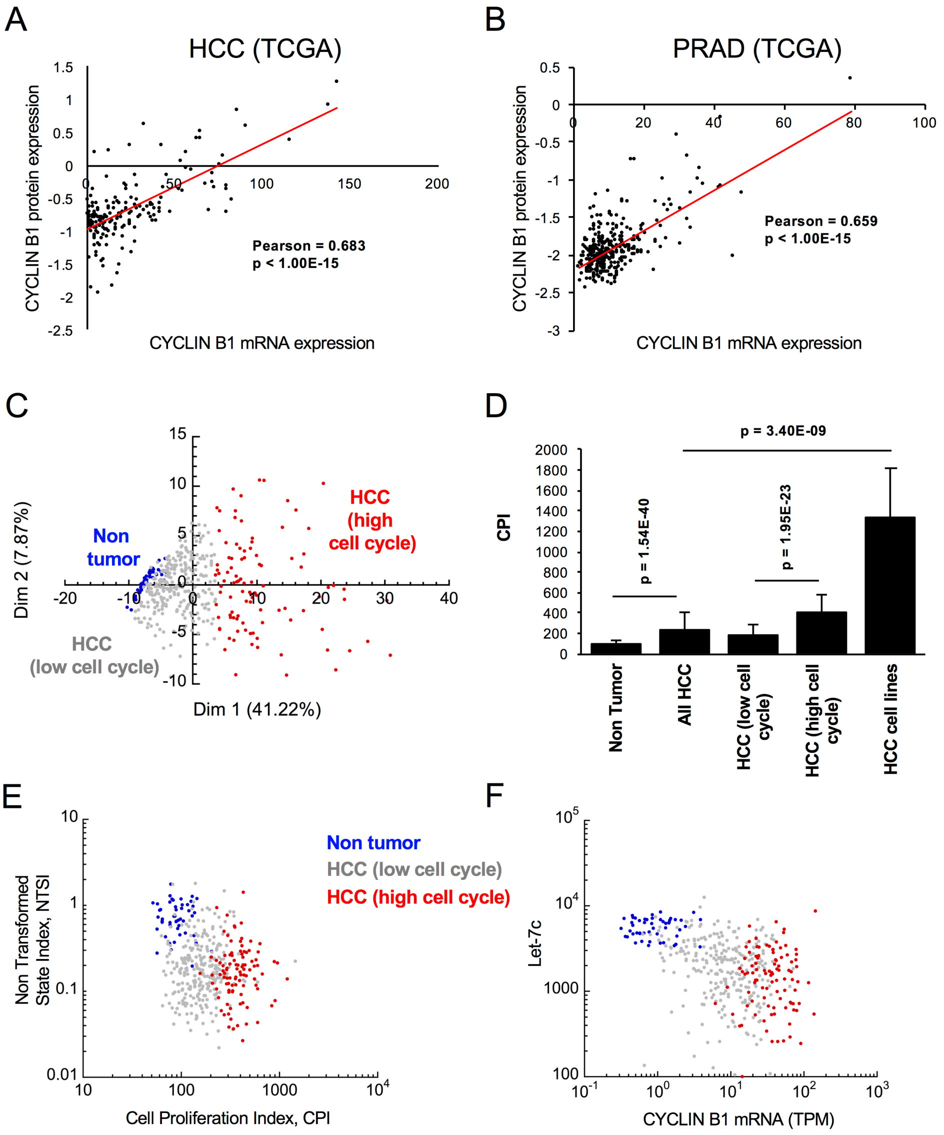

Figure S6. CPI correlates with cell cycle activation. (A, B) Expression levels of CYCLIN B1 protein (RPPA data, level 4, from the TCPA) as a function of CYCLIN B1 mRNA (TPM from TCGA) in the HCC cohort (A) and in prostate adenocarcinoma cohort (B). Each dot represents one sample in the cohort. For HCC cohort, n = 177, while for PRAD cohort, n = 348. (C) PCA analysis based on the mRNA expression of the cell cycle network components defined by KEGG. Blue dots : non tumor samples (n = 50), grey dots : HCC with low cell cycle activation (n = 268), red dots : 100 HCC samples with high cell cycle activation. (D) Mean value +/- SD of CPI in non tumor (n = 50), in all HCC (n = 368), in HCC low (n = 268) or high cell cycle activation (n = 100), and in 20 HCC cell lines (http://www.oasis-genomics.org/). (E, F) Expression levels of NTSI *versus* CPI and Let-7c *versus* *CYCLIN B1* mRNA in the HCC cohort.

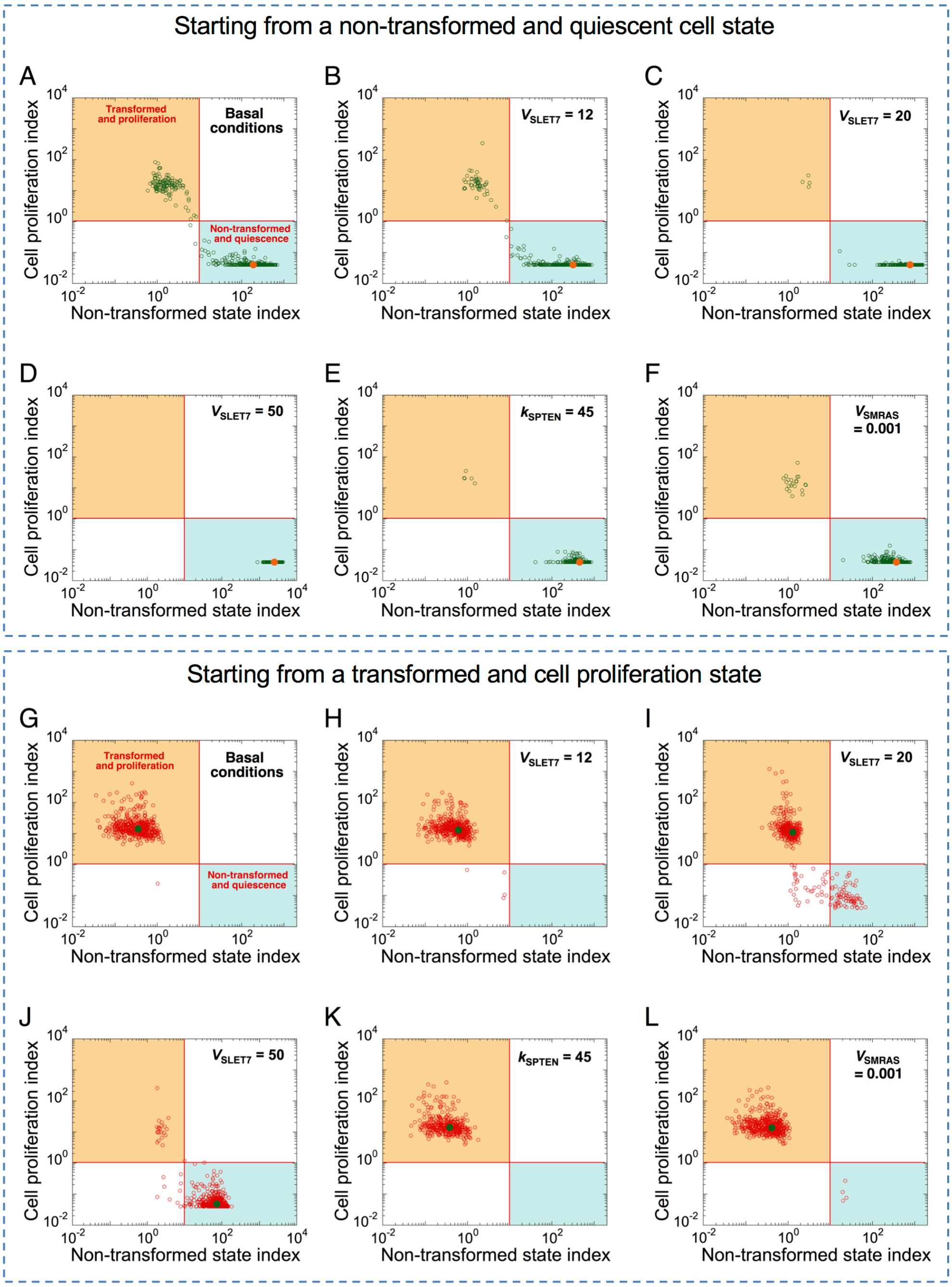

Figure S7. Oncogenes and tumor suppressors control the robustness of the networks dynamics. Starting from a non-transformed and quiescent cell state (A-F), or from a transformed and proliferating cell state (G-L), the cell proliferation index *versus* non-transformed state index is shown in a heterogeneous cell population with 25% of uniformed random variation of all parameters of the model. In both cases, simulations are performed in the basal conditions (A and G); in the presence of various overexpression levels of Let-7: V_{SLET7} = 12 in B and H, 20 in C and I, and 50 in D and J; in the presence of PTEN overexpression, k_{SPTEN} = 45 in E and K, or in the presence of Ras inhibition, V_{SMRAS} = 0.001 instead of 0.006 in F and L. In each case, circles correspond to one cell in a population of 500 cells. The orange (A-F) and green dots (G-L) correspond to the levels of *CPI* and *NTSI* in the absence of random variation on parameters. Basal condition corresponds to the parameter values in Table S5.

Supplementary references

Alexiades, M.R., and Cepko, C. (1996). Quantitative analysis of proliferation and cell cycle length during development of the rat retina. *Dev Dyn* 205**,** 293-307.

Gerard, C., and Goldbeter, A. (2011). A skeleton model for the network of cyclin-dependent kinases driving the mammalian cell cycle. *Interface Focus* 1**,** 24-35.

Gerard, C., Gonze, D., Lemaigre, F., and Novak, B. (2014). A model for the epigenetic switch linking inflammation to cell transformation: deterministic and stochastic approaches. *PLoS Comput Biol* 10**,** e1003455.

Goldbeter, A., and Koshland, D.E., Jr. (1981). An amplified sensitivity arising from covalent modification in biological systems. *Proc Natl Acad Sci U S A* 78**,** 6840-6844.

Iliopoulos, D., Hirsch, H.A., and Struhl, K. (2009). An epigenetic switch involving NF-kappaB, Lin28, Let-7 MicroRNA, and IL6 links inflammation to cell transformation. *Cell* 139**,** 693-706.

Le, S.J., J.; Husson, F. (2008). FactoMineR: An R Package for Multivariate Analysis. *Journal of Statistical Software* 25**,** 1-18.

Schwanhausser, B., Busse, D., Li, N., Dittmar, G., Schuchhardt, J., Wolf, J., Chen, W., and Selbach, M. (2011). Global quantification of mammalian gene expression control. *Nature* 473**,** 337-342.

Tyson, J.J., Chen, K.C., and Novak, B. (2003). Sniffers, buzzers, toggles and blinkers: dynamics of regulatory and signaling pathways in the cell. *Curr Opin Cell Biol* 15**,** 221-231.

Wagner, A. (2005). Circuit topology and the evolution of robustness in two-gene circadian oscillators. *Proc Natl Acad Sci U S A* 102**,** 11775-11780.

Wu, L., Fan, J., and Belasco, J.G. (2006). MicroRNAs direct rapid deadenylation of mRNA. *Proc Natl Acad Sci U S A* 103**,** 4034-4039.

Zhang, Z., Qin, Y.W., Brewer, G., and Jing, Q. (2012). MicroRNA degradation and turnover: regulating the regulators. *Wiley Interdiscip Rev RNA* 3**,** 593-600.
